# Genomic insights into historical population dynamics, local adaptation, and climate change vulnerability of the East Asian Tertiary relict *Euptelea* (Eupteleaceae)

**DOI:** 10.1111/eva.12960

**Published:** 2020-04-13

**Authors:** Ya‐Nan Cao, Shan‐Shan Zhu, Jun Chen, Hans P. Comes, Ian J. Wang, Lu‐Yao Chen, Shota Sakaguchi, Ying‐Xiong Qiu

**Affiliations:** ^1^ Systematic & Evolutionary Botany and Biodiversity Group MOE Laboratory of Biosystem Homeostasis and Protection College of Life Sciences Zhejiang University Hangzhou China; ^2^ College of Plant Protection Henan Agricultural University Zhengzhou China; ^3^ Department of Biosciences University of Salzburg Salzburg Austria; ^4^ Department of Environmental Science, Policy, and Management University of California Berkeley Berkeley CA USA; ^5^ Graduate School of Human and Environmental Studies Kyoto University Kyoto Japan

**Keywords:** East Asia’s Tertiary relicts, *Euptelea*, genomic vulnerability, historical population dynamics, local adaptation, restriction site‐associated DNA sequencing (RADseq)

## Abstract

The warm‐temperate and subtropical climate zones of East Asia are a hotspot of plant species richness and endemism, including a noticeable number of species‐poor Tertiary relict tree genera. However, little is understood about when East Asian Tertiary relict plants diversified, how they responded demographically to past environmental change, and to what extent their current genomic composition (and adaptive capacity) might mitigate the effects of global warming. Here, we obtained genomic (RAD‐SNP) data for 171 samples from two extant species of *Euptelea* in China (24 *E. pleiosperma* populations) and Japan (11 *E. polyandra* populations) to elucidate their divergence and demographic histories, genome‐wide associations with current environmental variables, and genomic vulnerability to future climate change. Our results indicate that Late Miocene changes in climate and/or sea level promoted species divergence, whereas Late Pliocene uplifting in southwest China likely fostered lineage divergence within *E. pleiosperma*. Its subsequent range expansion into central/east (*CE*) China bears genomic signatures of climate‐driven selection, yet extant *CE* populations are predicted to be most vulnerable to future climate change. For *E. polyandra*, geography was the only significant predictor of genomic variation. Our findings indicate a profound impact of Late Neogene geological and climate change on the evolutionary history of *Euptelea*, with much stronger signals of local adaptation left in China than in Japan. This study deepens our understanding of the complex evolutionary forces that influence the distribution of genetic variation of Tertiary relict trees, and provides insights into their susceptibility to global change and potential for adaptive responses. Our results lay the groundwork for future conservation and restoration programs for *Euptelea*.

## 1 INTRODUCTION

The warm‐temperate and subtropical climate zones of China and south/central Japan are a hotspot of plant biodiversity in East Asia (Qian & Ricklefs, 2000; Qiu, Fu, & Comes, 2011; Wang, 1992). In both regions, habitats associated with mountain riparian forests (MRFs) in particular feature high levels of species richness and endemism, including a noticeable number of species‐poor Tertiary relict tree genera (e.g., *Cercidiphyllum*, *Euptelea*, *Eurycorymbus*; Tang & Ohsawa, 2002; Wei et al., 2009). The MRF is also among the most threatened of all forest types in East Asia because of its high susceptibility to natural or human‐mediated erosion (Wei et al., 2009). The evolutionary and population demographic history of East Asia's Tertiary relict flora (*Cercidiphyllum*: Qi et al., 2012), or components of its affiliated MRF community (e.g., Meng, Wang, & Wang, 2016; Sun et al., 2011; Xing & Ree, 2017), has previously been associated with environmental changes since the Late Miocene (including changes in climate, topography, drainage systems, and sea level) using traditional molecular markers (e.g., DNA Sanger sequencing and microsatellites). For instance, *Cercidiphyllum japonicum* (Qi et al., 2012) has been cited as “more dynamic in history than previously thought” (cf. Mao & Liu, 2012), and the same is true for other Tertiary relicts showing a similarly wide geographic distribution (e.g., *Euptelea*: Cao, Comes, Sakaguchi, Chen, & Qiu, 2016; see below). However, given their limited number of variable loci, previous studies using traditional markers might be inadequate for characterizing the current genomic composition of species with complex demographic histories (Bay et al., 2018; Hancock et al., 2011). In consequence, further studies are required to disentangle the relative roles of historic‐environmental (geographical, climatic) and contemporary factors (e.g., drift, gene flow) in shaping the genomic architecture of East Asia's Tertiary relict tree species.

Such Tertiary relicts apparently persisted over long periods of geological time (in line with the concept of “living fossils”; Lidgard & Love, 2018). Moreover, in view of their constantly changing and often isolated MRF habitats (Wei et al., 2009), populations of these relicts are predicted to show signs of genetic impoverishment due to drift and/or limited gene exchange, thereby increasing their vulnerability to ongoing climate change (Bay et al., 2018; Yannic et al., 2014). This raises the question of how population genetic diversity relates to their alleged potential to endure environmental changes through adaptation (see also Parmesan, 2006; Parmesan & Yohe, 2003; Wiens, 2016; Yannic et al., 2014). In this era of rapid anthropogenic climate change, exploring such relationships is crucial to improve predictions of species’ climate vulnerability (Bay et al., 2018; Hoffmann & Sgro, 2011) and inform future conservation and restoration programs (Landguth et al., 2014; Ruegg et al., 2018).

In recent years, high‐throughput sequencing technologies, such as restriction site‐associated DNA sequencing (RADseq), have made it possible to rapidly collect genomic data and abundant single nucleotide polymorphisms (SNPs) in nonmodel organisms with increasing reliability and without prior information of a reference genome (Savolainen, Lascoux, & Merilä, 2013). When combined with approximate Bayesian computation (ABC), the RADseq approach in particular has proven useful in facilitating assessments of complex genetic structures and key demographic parameters, such as times since population isolation, postdivergence admixture rates, or changes in effective population size through time (e.g., Parchman, Jahner, Uckele, Galland, & Eckert, 2018). Moreover, the integration of RADseq and environmental (geographical, climatic) data provides exciting opportunities to identify population genomic diversity associated with current local adaptation or even vulnerability to future climate change (Bay et al., 2018; Fitzpatrick & Keller, 2015; Landguth et al., 2014; Ruegg et al., 2018).

In this study, we apply the above analytical approaches to RADseq data of *Euptelea* Sieb. et Zucc. (Eupteleaceae), one of the most widespread Tertiary relict tree genera of East Asia's MRF habitats (Sakai, Ohsawa, & Ohsawa, 1995; Wei, Jiang, Huang, Yang, & Yu, 2010; Wei, Meng, & Jiang, 2013). This genus has extensive fossil records throughout the Northern Hemisphere, extending to at least the Palaeocene (Manchester, Chen, Lu, & Uemura, 2009); today, however, it comprises just two extant species, *E. pleiosperma* Hook. f. et Thoms. and *E. polyandra* Sieb. et Zucc. (Cao et al., 2016). The distribution of *E. pleiosperma* extends from the southeastern margins of the Qinghai–Tibetan Plateau (QTP)/Hengduan Mts. Region (HMR) to central/east China, with populations occurring in isolated stands of MRF across a wide range of altitudes (*c*. 700–3,600 m above sea level). By contrast, *E. polyandra* is restricted to south/central Japan where it occurs in similar habitats of lower altitude (*c*. 100–1,600 m above sea level) (Sakai et al., 1995). Surprisingly, both species are still classified as “Least Concern” by the IUCN (International Union for Conservation of Nature; https://www.iucnredlist.org), although it has long been recognized that they are at risk of loss of their MRF habitats (Sakai et al., 1995; Wei et al., 2009).

Overall, this study aims to further clarify (a) when and how the two extant species of *Euptelea* diverged; (b) how they responded demographically to past environmental change; (c) to what extent historical, geographical, and/or climatic factors contribute to their current genomic variation; and (d) which populations of *E. pleiosperma* might be most vulnerable to future climate change. Hence, for comparison with our earlier study using plastid/nuclear DNA sequences and nSSR loci (Cao et al., 2016), our first objective was to use ABC simulations to determine the best model of population divergence and demographics that fits the patterns of RAD‐SNP diversity observed in *E. pleiosperma/E. polyandra*. Our second objective was to use a generalized dissimilarity model (GDM) framework (Manion et al., 2018) to explore the relative importance of environmental (geographical, climatic) factors underlying the neutral genomic variation of each species. In addition, by means of “*F*_ST_ outlier” tests, we scanned their genomes for signatures of climate‐driven local adaptation. Finally, we adopted a gradient forest (GF) approach (Ellis, Smith, & Pitcher, 2012) to predict the genomic composition of *Euptelea* populations under current and future (2050) climate scenarios (Fitzpatrick & Keller, 2015). Together, our results provide robust inferences about the historical population dynamics and adaptive capacity of an emblematic East Asian Tertiary relict genus, along with predictions of its “genomic vulnerability” to future climate change (sensu Fitzpatrick & Keller, 2015).

## 2 MATERIALS AND METHODS

### 2.1 Study system and sample collection

**Figure 1:**
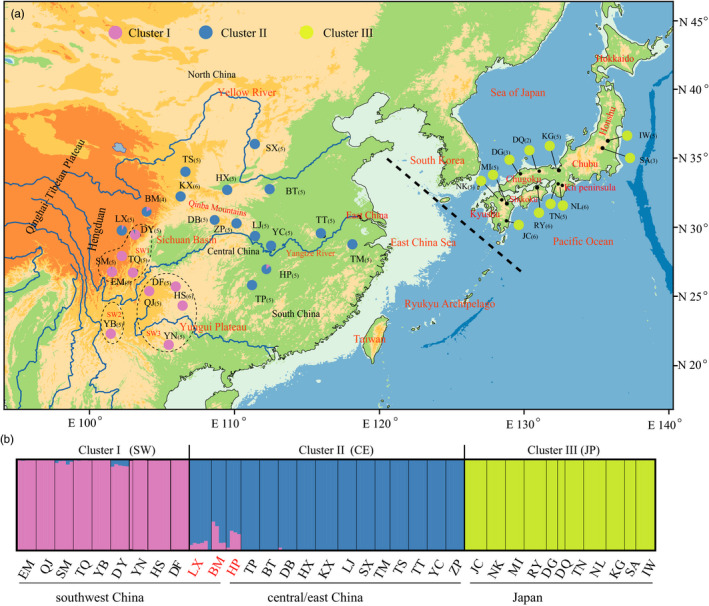
(a) Geographical distribution of three genomic clusters (I–III) identified by faststructure across 35 populations of *Euptelea* [24 of *E. pleiosperma* (China, *n* = 120) and 11 of *E. polyandra* (Japan, *n* = 51)] based on all three SNP datasets (data only shown for the “minimum” dataset). The black dashed line represents the species’ boundary across the East China Sea. The black dashed circles delimitate the three subclusters (*SW*1–3) revealed by DAPC for populations from west of the Sichuan Basin (cluster I). Numbers in parentheses represent the number of sequenced individuals per population. (b) Histogram of the faststructure analysis for *Euptelea* with *K* = 3. Each vertical bar represents one individual. Each cluster is represented by a distinct color

The two extant species of *Euptelea* are diploid (2*n* = 28), small‐ to medium‐sized (*c*. 2–15 m), broad‐leaved deciduous trees with bi‐sexual, wind‐pollinated flowers that develop into winged fruitlets (“samaras”), dispersed by gravity, wind, and/or water (see Cao et al., 2016, and references therein). For the RADseq analyses, we sampled 24 populations of *E. pleiosperma* (China, *n* = 120) and 11 of *E. polyandra* (Japan, *n* = 51), with 2–6 individuals per population (Figure 1, Table S1). Sample sizes in this range have been shown to produce reliable estimates of population demographics and divergence under a range of scenarios as long as enough loci (> 1,000) are investigated (Nazareno, Bemmels, Dick, & Lohmann, 2017; Robinson, Bunnefeld, Hearn, Stone, & Hickerson, 2014). This collection represents all major phylogeographic lineages identified in the genus based on previous chloroplast (cp) DNA and nuclear microsatellite (nSSR) data (Cao et al., 2016).

### 2.2 RADseq data acquisition, processing, and SNP genotyping

RAD libraries were prepared and sequenced for each DNA sample (in total 171 samples) by Beijing Genomics Institute (BGI; Shenzhen, China) using the restriction enzyme *EcoR*I and sample‐specific barcodes. The individuals in the libraries were pooled and run on two lanes of Illumina HiSeq 2500 to generate 150‐bp paired‐end reads. We demultiplexed and processed Illumina reads using the software pipeline ipyrad v0.4.7 (Eaton, 2014; Eaton & Ree, 2013). Any RAD reads containing sequencing errors in the sample‐specific barcodes and restriction cut sites were removed. Nucleotide bases with a Phred quality score (*Q*) below 33 were replaced with an ambiguous base (“N”), and reads with more than 10% “N”s were discarded. Filtered reads of each individual were first assembled de novo into putative loci. For within‐sample clustering, sequences were clustered at 90% similarity. In order to ensure accurate base calls, only clusters that had a minimum depth of coverage ≥ 6 were retained. After clustering, error rate (*E*) and heterozygosity (*H*) were jointly estimated from the base counts in each site across all aligned clusters for each sampled individual (Lynch, 2008), and the average parameter values were used when calling consensus bases. Bases that could not be assigned with ≥ 95% probability in the consensus sequences were replaced with appropriate ambiguity code (N) in the consensus sequence. In addition, loci containing more than two alleles after error correction were excluded as potential paralogs since both *Euptelea* species are diploid. Consensus sequences were then clustered across samples at 90% similarity and aligned with muscle v3.8.31 (Edgar, 2010). A final filtering step excluded loci that contain any site appearing heterozygous across more than 25% of samples (Table S2), as this is more likely to represent a fixed difference among clustered paralogs than a true polymorphism (Hohenlohe, Amish, Catchen, Allendorf, & Luikart, 2011). The remaining clusters representing multiple alignments of putative orthologs were treated as RADseq loci and assembled into population genomic data matrices. We kept only one SNP per RADseq locus to create a dataset without closely linked loci.

To explore the effect of missing data (locus dropout or low coverage) and ensure enough SNPs for analyses, we assembled three data matrices with different minimums for sample coverage (the number of samples for which data must be recovered to include a RAD locus in the dataset): (a) the data matrix that includes all loci shared across at least 85 samples (“maximum” dataset); (b) the median data matrix that contains all loci shared across at least 120 samples (“median” dataset), and (c) the data matrix that includes all loci shared across at least 160 samples (“minimum” dataset). Following a previous study's suggestion (Paris, Stevens, & Catchen, 2017), a locus was kept only if it occurred in at least 60% of samples within each population to ensure a wide representation of each SNP across all sampling sites. We designated a 1% minor allele frequency (MAF) cutoff to the three datasets. To evaluate whether our inference of population structure is robust to missing data and rare alleles, we performed population structure analyses using datasets with and without filtering loci with MAFs < 0.01.

### 2.3 Population genetic structure and diversity

Bayesian clustering of individuals was conducted for the three *Euptelea* datasets (“maximum,” “median,” and “minimum”) with and without filtering loci with MAFs < 0.01 using faststructure v1.0 (Raj, Stephens, & Pritchard, 2014). The number of clusters (*K*) was set to vary depending on the dataset. The most probable values of *K* for explaining population structure were determined by estimating the minimum value of *K* that accounts for almost all of the ancestry in the three datasets and maximizes the log marginal likelihood lower bound. In addition, for each of the three datasets, population structure was also investigated by discriminant analysis of principal components (DAPC) for *Euptelea* and each species, respectively, using the r package adegenet (Jombart, 2008). The optimal number of clusters was chosen on the basis of the lowest associated Bayesian information criterion (BIC). Finally, we subjected each of the three SNP matrices to a maximum likelihood (ML) tree inference analysis in raxml‐hpc v7.2.8 (Stamatakis & Ott, 2008) under the general time‐reversible (GTR) substitution model and with 11 individuals randomly selected from each *E. polyandra* population as outgroups and missing data coded as “N”s.

Based on the putatively neutral RAD‐SNP loci of the “minimum” dataset, that is, 8,733 SNPs (see Results), mean nucleotide diversity (*π*) and average expected and observed heterozygosities (*H*_exp_/*H*_obs_) were calculated for each population with *n* ≥ 5 using arlequin v3.5 (Excoffier & Lischer, 2010)*.* For *E. pleiosperma*, we also calculated *π*, *H*_exp_, and *H*_obs_ from only the 49 outlier loci identified (see below and Results). Five *Euptelea* populations (BM, TM, DG, DQ, and SA) with small sample size (*n* < 5) were removed from all population‐level analyses of genetic diversity (marked with asterisks in Table S1). For *E. pleiosperma* and *E. polyandra*, measures of genetic diversity (*π* and *H*_exp_/*H*_obs_) were regressed against latitude and longitude, respectively, using the ggplot2, iswr, and scales packages implemented in r v3.3 (R Development Core Team, 2015) to test the hypothesis that suites of environmental conditions could promote or constrain different levels of genetic diversity. Analysis of molecular variance (AMOVA) in arlequin was used to quantify the genomic variance among species and populations, with significance of *Φ*‐statistics tested using 10,000 permutations (Excoffier & Lischer, 2010).

### 2.4 ABC modeling of divergence and demographic histories

We used the coalescent‐based approximate Bayesian computation (ABC) implemented in diy‐abc v2.0 (Cornuet et al., 2014) to infer the divergence and demographic histories of *Euptelea*. To avoid the impacts of missing data and removal of rare alleles on the inference of population history, we performed our ABC analysis on a high‐quality data matrix without missing data and without filtering loci with MAFs < 0.01 (the “full” dataset, i.e., 1,383 SNPs) across all 171 samples, as the two extant *Euptelea* species apparently underwent climate‐induced expansions (Cao et al., 2016; Wei, Sork, Meng, & Jiang., 2016) and were thus expected to have an excess of rare alleles (Excoffier, Dupanloup, Huerta‐Sánchez, Sousa, & Foll., 2013). We tested five plausible divergence scenarios on the basis of the genetic structure identified by faststructure and DAPC (see Results): the simultaneous divergence of three regional groups (i.e., southwest China: *SW*; central/east China: *CE;* and Japan: *JP*) from a common ancestor (Figure S1a, Scenario 1) against three alternative models, reflecting all possible relationships among these groups (see Scenarios 2–4 in Figure S1a), and an admixture model, in which the *SW* and *JP* groups diverged from an ancestral population at time *t*_1_, followed by an admixture event between them at time *t*_2_, which then gave rise to the *CE* group with admixture rate *ra* (Scenario 5 in Figure S1a). We selected all summary statistics (Table S3) of genetic variation to generate reference tables with 10^6^ simulated datasets for the five models. The parameters describing each model (i.e., divergence times, admixture rate, and effective population sizes; Table 3) were treated as random variables drawn from uniform prior distributions with a restriction on temporal parameters (*t*_1_ > *t*_2_).

First, we performed leave‐one‐out cross‐validation using neural network method for model selection via the “cv4postpr” function in the “abc” R package to evaluate whether model selection with ABC is able to distinguish between the five proposed models by making use of the existing simulations from diy‐abc (Csilléry, François, & Blum., 2012). Next, we calculated the posterior probabilities of each demographic scenario using the multinomial logistic regression and neural network methods implemented with the function “postpr” across a range of tolerances (0.001, 0.005, 0.001, 0.05) (Csilléry et al., 2012). The goodness of fit of the scenario with the highest posterior probability was assessed using the “model checking” option with principal component analysis (PCA) in diy‐abc, which evaluated the discrepancy between the model and the observed data (Tsuda, Nakao, Ide, & Tsumura., 2015). We then used the function “cv4abc” to evaluate the accuracy of ABC parameter estimates and the robustness of the estimates to tolerance rates. The accuracy of parameter estimates was evaluated under tolerance rates of 0.001, 0.005, and 0.01 using the rejection, the local linear regression, and neural network methods. Finally, because of a lower prediction error rate (see results), a local linear regression was used to estimate the posterior distributions of parameters for the best‐fitting scenario on 1% of the simulated datasets closest to the observed dataset and applying a *logit* transformation to parameter values (Beaumont, Zhang, & Balding, 2002). To convert estimated divergence times into millions of years ago, we assumed a conservative generation time of 10 years for *Euptelea* (Sakai et al., 1995).

Additionally, we used diy‐abc to investigate past changes in population size in each regional lineage of *E. pleiosperma* (*SW* and *CE*) and *E. polyandra*. We tested three simple models of population size changes: (a) population growth following a constant population size (“expansion”); (b) expansion followed by shrinkage (“shrinkage”); and (c) expansion followed by shrinkage and a new expansion event (“expansion‐shrinkage‐expansion”) (Figure S1b; Wang et al., 2016). We used the same strategies as detailed above to choose the demographic scenarios that best fit the data and estimated the parameters of interest.

### 2.5 Generalized dissimilarity model (GDM) of genomic, geographic, and climatic data

To evaluate the effects of geographic distance and environmental dissimilarity on genetic differentiation, we fit generalized dissimilarity models (GDMs; Manion et al., 2018) to our “minimum” dataset (8,782 RAD‐SNPs) (see Results) for the 22 *E. pleiosperma* populations (*n* ≥ 5) and the eight *E. polyandra* populations (*n* ≥ 5), respectively. GDM is a nonlinear extension of matrix regression that models spatial patterns of pairwise genetic dissimilarity between sampling sites caused by pairwise site differences in environmental and geographic variables (Fitzpatrick & Keller, 2015). For each species, we constructed three site‐by‐environment predictor matrices from values of 19 bioclimatic variables (Table S4) extracted at each locality from GIS data layers at 30 arc‐sec resolution (1960–1990) that we downloaded from WorldClim (http://www.worldclim.org). To retain only the predictors that significantly contributed to the model in each GDM analysis, we employed a backward elimination procedure (Ferrier, Manion, Elith, & Richardson, 2007). Starting with the full model, this process iteratively removes the variable with the lowest coefficient, recalculates the model fit, and uses a variable permutation procedure to assess significance. Under the permutation procedure, the significance of the model is tested by permuting all predictor variables, refitting the model under each permutation to generate a null distribution of deviance explained values, and then comparing the data‐driven model to the null distribution. The significance of each predictor variable is tested by permuting each variable individually to generate a null distribution of the change in deviance explained for the model and comparing each variable's contribution to the model against the null distribution. The final outcome is a fitted model that retains only the statistically significant predictor variables (Manion et al., 2018).

Based on the 8,733 neutral RAD‐SNPs of our “minimum” dataset, we generated two response matrices of AMOVA‐derived *Φ*_ST_ values between pairs of populations for the 22 *E. pleiosperma* populations (*n* ≥ 5) and the eight *E. polyandra* populations (*n* ≥ 5) (Table S1), respectively, using arlequin. Then, we fit GDMs to the response and predictor matrices and used the resulting models to explore the spatial and climatic drivers of differences in genetic turnover. To estimate the relative genetic importance of each predictor, we adjusted the maximum values of the fitted I‐splines to a range from −1.5 to 1.5. We used the r package gdm (Manion et al., 2018) to fit models and assessed model performances by computing percent deviance explained.

### 2.6 Detecting signatures of climate‐driven local adaptation

We scanned the “minimum” dataset for outlier loci in *E. pleiosperma* and *E. polyandra* (populations with *n* ≥ 5; Table S1), respectively, using the Bayesian approach implemented in bayescan v2.1 (Foll & Gaggiotti, 2008) and the nonhierarchical model implemented in arlequin (Excoffier & Lischer, 2010). As a fully Bayesian approach, bayescan directly estimates the posterior probability that each locus is under selection by decomposing locus‐population *F*_ST_ coefficients into a locus‐specific component (alpha) shared by all populations and a population‐specific component (beta) shared by all loci. We ran the program bayescan with the following settings: 5,000 iterations; 20 thinning intervals; 20 pilot runs of length 5,000; 50,000 additional burn‐in; uniform distribution between 0 and 1; and a prior odd of 10 for neutral model. Positive values of alpha indicate diversifying selection, whereas negative values indicate balancing or purifying selection (Foll & Gaggiotti, 2008). *Q*‐values of the loci were also automatically calculated by the program, and those results (alpha > 0) were filtered to retain loci with *q*‐values below 0.001. For the hierarchical model in arlequin, 20,000 simulations were conducted with 100 demes per population, with the false discovery rate (FDR) set at 0.01. Loci bearing signatures of diversifying selection identified by both methods were segregated into an outlier matrix, and the remaining loci without outliers constituted the neutral dataset.

For the 49 outlier loci detected in *E. pleiosperma* (see Results), we calculated population allele frequencies in arlequin and used multiple linear regressions (MLRs; Zulliger, Schnyder, & Gugerli, 2013) to test for their association with the six variables most important in explaining the observed genetic variation in the GDM (Table S4). Bioclimatic values per site were extracted as in the GDM (see above). All regressions were performed using the r package vegan v2.5.1 (Oksanen et al., 2018). Loci showing model fit (*R* ^2^_adj_) values > 0.5 and significant correlation with at least one variable were considered “adaptive” loci (Manel, Poncet, Legendre, Gugerli, & Holderegger, 2010). Lastly, we used the r package gradient forest (Ellis et al., 2012) to investigate patterns of allelic turnover at each of the 49 outlier loci with regard to the six variables.

### 2.7 Gradient forest prediction of genomic vulnerability to future climate change

We further used gradient forest to predict *Euptelea's* “genomic vulnerability” using the method proposed by Fitzpatrick and Keller (2015). Here, “genomic vulnerability” is a measure of the mismatch between genotypes and future predicted environment using associations across contemporary climate gradients as a baseline. The current (1960–1990) and future (based on 2050 RCP2.6 projections, Van Vuuren et al., 2012) bioclimatic variables (Table S4) were downloaded from WorldClim. For the implementation of the gradient forest model, we first calculated population allele frequencies from the all 8,782 SNPs loci of our “minimum” dataset and extracted current bioclimatic variables for each population (parameter settings: 500 regression trees per SNP; maxLevel = log2(0.368n)/2; variable correlation threshold: 0.5). The fitted model was then used to predict genomes under current and future climate scenarios across the entire range of the genus by projecting the model onto the future climate layers. For each grid cell, “genomic vulnerability” was calculated as the Euclidian distance between current and predicted genomic compositions (Fitzpatrick & Keller, 2015). Lastly, we mapped this Euclidian distance metric at the genus’ range‐wide scale (using ecological niche distribution models of Cao et al., 2016) to visualize regions (and populations) predicted to experience greater impacts under future (2050) compared to current climate conditions.

## 3 RESULTS

### 3.1 RADseq data and processing

A total of *c.* 1,380 million reads passed quality checking. After quality filtering, the number of reads per sample averaged 8.08 × 10^6^ (minimum: 1.63 × 10^6^; maximum: 19.35 × 10^6^) with an average read depth of 28.78 (range: 11.17–60.68) (Table S5). For each individual, the assembled RAD clusters (or “stacks”) with a sequence similarity threshold of 90% ranged from 0.86 × 10^5^ to 3.26 × 10^5^. The number of consensus sequences called for each cluster averaged 1.93 × 10^5^ (range: 0.72–2.75 × 10^5^). Clustering of consensus sequences across all 171 samples by ipyrad yielded 29,494 informative sites (unlinked SNPs) for the “minimum” dataset, 89,158 for the “median” dataset, and 107,839 for the “maximum” dataset. After filtering loci with excess missing data within population, 18,182 (“minimum” dataset), 75,101 (“median” dataset), and 76,365 (“maximum” dataset) SNPs were retained, of which 51.70%, 48.71%, and 48.69% SNPs had a MAF ≤ 1% (Figure S2), respectively. Our final MAF‐filtered datasets retained 8,782 informative sites for the “minimum” dataset, 38,522 for the “median” dataset, and 39,180 for the “maximum” dataset. A total of 8,733 loci in the “minimum” dataset passed the two filtering steps for neutrality (see below).

### 3.2 Population genetic structure and diversity

**Figure 2:**
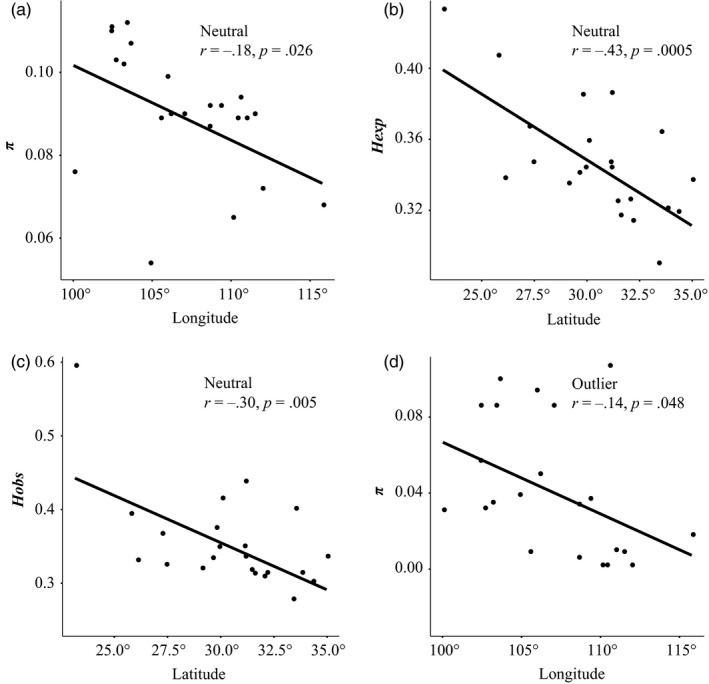
Relationship between latitude/longitude and genetic diversity measures (*π*: nucleotide diversity; *H*_exp_: average expected heterozygosity; *H*_obs_: average observed heterozygosity) for *E. pleiosperma* populations based on 8,733 neutral RAD‐SNPs (a: *π*; b: *H*_exp_; c: *H*_obs_) and 49 outliers (d: *π*)

For the RAD‐SNPs from the three datasets with or without filtering loci with MAFs < 0.01 (35 populations, *n* = 171), genetic structure analysis in faststructure (Figure 1) consistently provided support for a three‐cluster model. Within *E. pleiosperma* (China), most populations located west versus east of the Sichuan Basin were assigned to clusters I (“southwest China”: *SW*) versus II (“central/east China”: *CE*); as an exception, two populations from northwest of the basin (BM, LX) and a population from southeast of the basin (HP) clearly belonged to cluster II as well, while showing traces of admixture with the *SW* lineage (Figure 1). By contrast, individuals of *E. polyandra* (Japan) exclusively formed a distinct cluster (III). Separate faststructure analyses of *E. pleiosperma* and *E. polyandra* resulted in similar patterns. For each of the datasets, the DAPC for *Euptelea* (Figures S3a, S4a) identified the same clusters (I–III) at *K* = 3 (i.e., the optimal value based on BIC; Figure S5a), while a separate DAPC on *E. pleiosperma* (Figures S3b, S4b) further divided its *SW* lineage into three subclusters (*SW*1‒3) at *K* = 4 (the optimal solution; Figure S5b). The topology of the ML tree based on the “maximum” dataset (Figure S6b) was identical to that estimated from the “median” data and was quite similar to that based on the “minimum” data (Figure S6a). In the rooted trees (Figure S6), samples of *E. polyandra* formed a monophyletic clade (bootstrap percentage, BP = 100%), and those of *E. pleiosperma* were also monophyletic (BP = 100%), with two subclades (each 100%) representing the *SW* versus *CE* lineages, respectively. Within the latter group, all samples from east of the Sichuan Basin (except for HP) occupied a nested position relative to those from the northwest (BM/LX) and southeast (HP). The AMOVA based on the neutral “minimum” dataset (Table 1) revealed that genetic differentiation among populations was much higher in *E. pleiosperma* (*Φ*_ST_ = 0.44) than in *E. polyandra* (*Φ*_ST_ = 0.13), and *c*. 69.74% of the total genetic variation resided among species (*Φ*_CT_ = 0.70). In *E. pleiosperma*, the *SW* lineage showed much stronger population differentiation (*Φ*_ST_ = 0.27) than the *CE* lineage (*Φ*_ST_ = 0.13; Table 1). *E. polyandra* had average higher levels of neutral (i.e., based on 8,733 SNPs) within‐population genetic diversity than those of *E. pleiosperma* (*E. polyandra/E. pleiosperma*: *π* = 0.203 vs. 0.089, *H*_exp_ = 0.364 vs. 0.346; Table S1). Within *E. pleiosperma*, *SW* and *CE* harbored similar levels of diversity (*SW/CE*: *π* = 0.095/0.085, *H*_exp_ = 0.360/0.336). Moreover*,* neutral within‐population estimates of *π* in this species significantly decreased with longitude (*r* = −0.18, *p = *.026; Figure 2a), as was the case for *H*_exp_ and *H*_obs_ with regard to latitude (*H*_exp_/ *H*_obs_:* r* = −0.43/‐0.30, *p = *.0005/0.005; Figure 2b,c); no such associations were found between *π* and latitude (*r* = 0.005, *p = *.35), or between *H*_exp_/*H*_obs_ and longitude (*r* = 0.05/0.035, *p = *.91/0.60). In *E. polyandra*, all three diversity measures (*π*, *H*_exp_, and *H*_obs_) were independent of latitude and longitude (all *p* values > .05).

**Table 1:** Analyses of molecular variance (AMOVAs) based on neutral RAD‐SNPs of the “minimum” dataset for *Euptelea*, *E. pleiosperma* and its two lineages (*SW* and *CE*), and *E. polyandra*

Source of variation	*df*	Percentage of total variance (%)	*Φ*‐statistics
*Euptelea*			
Among species	1	69.74	*Φ*_CT_ = 0.70*
Among populations within species	28	11.4	*Φ*_SC_ = 0.38*
Within populations	280	18.85	*Φ*_ST_ = 0.81*
*E. pleiosperma*			
Among populations	21	43.59	*Φ*_ST_ = 0.44*
Within populations	202	56.41	
*SW*			
Among populations	8	26.84	*Φ*_ST_ = 0.27*
Within populations	83	73.16	
*CE*			
Among populations	12	12.71	*Φ*_ST_ = 0.13*
Within populations	119	87.29	
*E. polyandra*			
Among populations	7	12.79	*Φ*_ST_ = 0.13*
Within populations	78	87.21	

* *p* < .05.

### 3.3 ABC‐based inference of divergence and demographic histories

**Figure 3:**
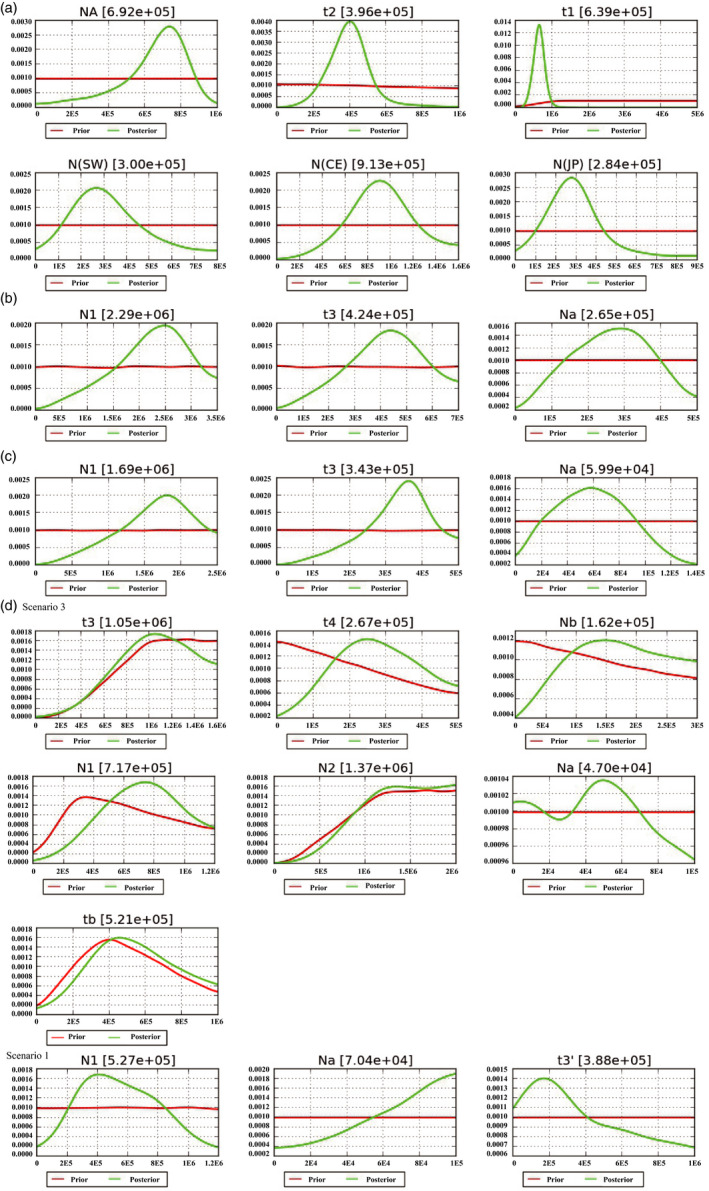
Estimations of the prior and posterior distribution of parameters revealed by DIY‐ABC modeling of the best‐fit scenarios for (a) divergence model and demographic history of (b) *SW* lineage, (c) *CE* lineage, and (d) *JP* lineage. See Table 3 for identification of corresponding parameter codes. The time parameters are estimated in generations and converted into years by multiplying generation time, which was set to 10 years for *Euptelea* species

**Figure 4:**
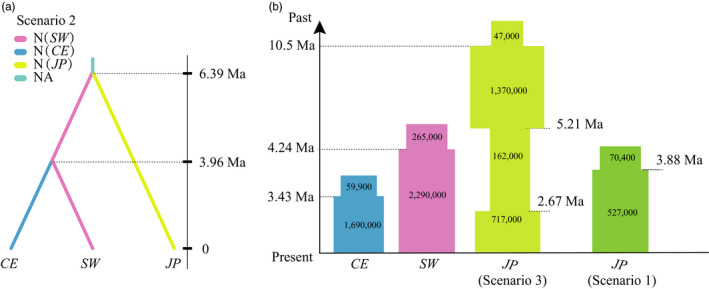
(a) The best ABC divergence model (Scenario 2) with divergence times for *Euptelea* based on diy‐abc analyses of RAD‐SNP data (i.e., 1,383 loci). The “ancestral population,” with an effective population size of NA, is represented in light blue. (b) Population size estimates through time for the two lineages of *E. pleiosperma* (*SW* and *CE*) and *E. polyandra* (*JP*) under the best‐fitting ABC demographic models. Times of divergence are indicated by horizontal dashed lines, assuming a generation time of 10 years (see text). Only the median values are shown (see Table 3 for 95% highest posterior density intervals for all values)

Assessments of the performance of ABC model selection analyses show that the simulated model was correctly identified in between 68% and 96% of cross‐validation replicates, demonstrating all scenarios were distinguished correctly by the calculated summary statistics for both divergence and demographic history inferences of *Euptelea* (Figure S7). When divergence history was examined using ABC based on the “full” dataset (i.e., 1,383 SNPs), “Scenario 2” (i.e., ancient divergence of *E. pleiosperma* and *E. polyandra* and more recent origin of *CE* from within the *SW* lineage of *E. pleiosperma*; Figures 3a, 4a) was the best fit to the data, as it had significantly higher posterior probability than the other four scenarios tested under the both multinomial logistic regression and neural network methods (Figure S1a and Table 2). Regarding demographic history, the best‐fit scenarios for both the *SW* and *CE* lineages of *E. pleiosperma* were Scenario 1 (“expansion”). For *E. polyandra*, although Scenario 3 (“expansion‐shrinkage‐expansion”) showed the highest posterior probability, Scenario 1 (“expansion”) was also found to have a high posterior probability (Figure S1b and Table 2). In the corresponding goodness‐of‐fit PCA graphs (divergence: Figure S8a; demography: Figure S8b–d), the observed data points were located within a large cluster of points for the simulated data from the prior and within a smaller cluster of data from the posterior predictive distribution, indicating good model performance.

**Table 2:** Model comparison in approximate Bayesian computation analysis. Posterior probability values and Bayes factors in brackets (of the best supported scenario against the respective model) are shown for the multinomial logistic regression and neural network methods

Methods	Multinomial logistic regression				Neural network			
Tolerance rate	0.001	0.005	0.01	0.05	0.001	0.005	0.01	0.05
Divergence model								
Scenario 1	0 (3.308e31)	0 (5.263e20)	0 (2.224e16)	0 (3.297e8)	0.037 (17.822)	0.033 (27.323)	0 (5.679e6)	0.001 (1.334e3)
Scenario 2	**1**	**1**	**1**	**0.998**	**0.662**	**0.895**	**0.906**	**0.912**
Scenario 3	0 (1.682e5)	0 (6.149e6)	0 (3.160e6)	0 (1.929e5)	0.099 (6.671)	0.030 (29.198)	0.012 (76.589)	0.073 (12.556)
Scenario 4	0 (2.533e10)	0 (3.110e11)	0 (1.281e13)	0 (2.618e10)	0.003 (215.742)	0.021 (42.642)	0 (2.222e3)	0.002 (413.683)
Scenario 5	0 (3.990e11)	0 (5.322e3)	0 (3.229e6)	0.002 (494.989)	0.198 (3.339)	0.021 (42.356)	0.082 (11.102)	0.012 (72.799)
Demographic model								
*E. pleiosperma (SW lineage)*								
Scenario 1	**0.872**	**0.871**	**0.863**	**0.820**	**0.874**	**0.872**	**0.876**	**0.873**
Scenario 2	0.049 (17.838)	0.051 (17.184)	0.052 (16.521)	0.058 (14.111)	0.046 (19.203)	0.049 (17.848)	0.048 (18.052)	0.049 (17.847)
Scenario 3	0.079 (11.012)	0.078 (11.128)	0.085 (10.195)	0.122 (6.741)	0.080 (10.927)	0.079 (11.069)	0.076 (11.513)	0.078 (11.135)
*E. pleiosperma (CE lineage)*								
Scenario 1	**0.840**	**0.837**	**0.828**	**0.811**	**0.850**	**0.840**	**0.839**	**0.835**
Scenario 2	0.080 (10.547)	0.088 (9.551)	0.092 (8.993)	0.102 (7.987)	0.077 (11.019)	0.084 (10.001)	0.086 (9.679)	0.089 (9.363)
Scenario 3	0.080 (10.431)	0.075 (11.173)	0.080 (10.417)	0.088 (9.250)	0.073 (11.712)	0.076 (11.047)	0.075 (11.234)	0.076 (11.067)
*E. polyandra (JP lineage)*								
Scenario 1	0.353 (1.440)	0.381 (1.281)	0.387 (1.253)	0.404 (1.151)	0.354 (1.449)	0.378 (1.297)	0.388 (1.245)	0.386 (1.269)
Scenario 2	0.139 (3.670)	0.13 (3.753)	0.129 (3.740)	0.131 (3.553)	0.133 (3.848)	0.132 (3.703)	0.128 (3.769)	0.125 (3.928)
Scenario 3	**0.508**	**0.489**	**0.484**	**0.465**	**0.513**	**0.49**	**0.484**	**0.489**

Note

The best scenario was marked in bold.

Cross‐validation for parameter estimation showed that local linear regression had a lower prediction error for most parameters when compared with the other two methods (i.e., rejection and neural network) (Table S6). Therefore, we calculated posterior distributions of all parameters using a local linear regression (Figure 3). According to Scenario 2, we dated the split between *E. pleiosperma* and *E. polyandra* at about the Late Miocene, *c.* 6.39 (95% CI: 3.90–8.92) Ma, and the origin of *E. pleiosperma's CE* lineage (from within the *SW* lineage) at about the Late Pliocene, *c.* 3.96 (95% CI: 2.22–5.93) Ma (Table 3; Figures 3a, 4a). In addition, the expansions of the *SW* and *CE* lineages (see above) were estimated to have occurred at *c.* 4.24 (95% CI: 1.52–6.48) Ma and 3.43 (95% CI: 1.47–4.68) Ma, respectively (Table 3; Figures 3b, 4b). For *E. polyandra*, the best‐fit Scenario 3 indicated that *E. polyandra* initially expanded up to the Early Pliocene, *c*. 5.21 (95% CI: 2.40–8.53) Ma, then experienced a long‐term (*c.* 2.50 Myr) reduction in population size (*c.* 8.5‐fold), and expanded again at the beginning of the Late Pliocene, *c*. 2.67 (95% CI: 0.83–4.65) Ma. By contrast, Scenario 1 indicated that the expansion of *E. polyandra* was estimated to have occurred at the Mid‐Pliocene, *c*. 3.88 (95% CI: 0.71–9.21) Ma (Table 3; Figures 3 and 4b).

**Table 3:** Descriptions of prior settings and median estimates of posterior distributions for all parameters in the best‐fitting scenarios based on diy‐abc

Parameters	Priors^a^	Posteriors		
		Median	95% lower bound	95% upper bound
Divergence model				
*Euptelea* (Scenario 2)				
NA	10‒1.00E + 06	6.92E + 05	2.44E + 04	8.50E + 05
*N*(*SW*)	10‒8.00E + 05	3.00E + 05	1.05E + 05	6.59E + 05
*N*(*CE*)	10‒1.60E + 06	9.13E + 05	4.46E + 05	1.42E + 06
*N*(*JP*)	10‒9.00E + 05	2.84E + 05	1.03E + 05	6.22E + 05
*t*_1_	100‒5.00E + 07	6.39E + 06	3.90E + 06	8.92E + 07
*t*_2_	100‒1.00E + 07	3.96E + 06	2.22E + 06	5.93E + 06
Demography model				
*SW* (Scenario 1)				
Na	10‒5.00E + 05	2.65E + 05	6.84E + 04	4.47E + 05
N1	10‒3.50E + 06	2.29E + 06	8.46E + 05	3.27E + 06
*t*_3_	100‒7.00E + 06	4.24E + 06	1.52E + 06	6.48E + 06
*CE* (Scenario 1)				
Na	10‒1.40E + 05	5.99E + 04	1.34E + 04	1.16E + 05
N1	10‒2.50E + 06	1.69E + 06	6.75E + 05	2.37E + 06
*t*_3_	100‒5.00E + 06	3.43E + 06	1.47E + 06	4.68E + 06
*E. polyandra* (Scenario 3)^b^				
Na	10‒1.00E + 05	4.70E + 04	5.19E + 03	9.46E + 04
N2	10‒2.00E + 06	1.37E + 06	6.52E + 05	1.94E + 06
Nb	10‒3.00E + 05	1.62E + 05	3.80E + 04	2.85E + 05
N1	10‒1.20E + 06	7.17E + 05	2.96E + 05	1.11E + 06
*t*_3_	100‒1.60E + 07	1.05E + 07	4.95E + 06	1.53E + 07
*t*_b_	100‒1.00E + 0)	5.21E + 06	2.40E + 06	8.53E + 06
*t*_4_	100‒5.00E + 06	2.67E + 06	8.25E + 05	4.65E + 06
*E. polyandra* (Scenario 1)^b^				
Na	10‒1.00E + 05	7.04E + 04	1.39E + 04	9.75E + 04
N1	10‒1.20E + 06	5.27E + 05	1.95E + 05	9.82E + 05
*t_3_’*	100‒1.00E + 07	3.88E + 06	7.14E + 05	9.21E + 06

^a^ All priors are uniformly distributed. *N*(*SW*), *N*(*CE*), and *N*(*JP*) denote the current effective population sizes of the *SW* and *CE* lineages of *E. pleiosperma* and *E. polyandra*, respectively (see Figure 4).

^b^ For demographic history of *E. polyandra,* Scenario 3 and Scenario 1 *have similar high* posterior probability. NA is the effective population size of the common ancestor of the three groups. Na: ancestral population size for each group; N1: current population size; N2 and Nb: population sizes between Na and N1; *t*_1_: divergence time between *E. pleiosperma* and *E. polyandra*; *t*_2_: divergence time between *SW* and *CE*; *t*_3_ and *t*_3_’: old expansion time; *t*_b_: bottleneck time; *t*_4_: recent expansion time.

### 3.4 Impact of geographical and climatic factors on genetic structure

**Figure 5:**
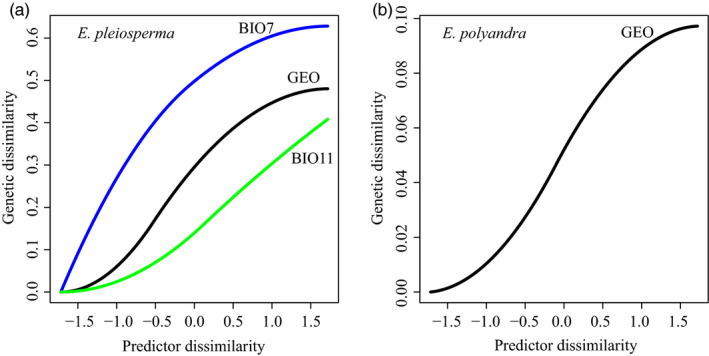
GDM‐fitted I‐splines of environmental and geographic distance for (a) *E. pleiosperma* and (b) *E. polyandra*, based on 8,733 neutral RAD‐SNPs. The maximum height of each spline indicates the total amount of allelic turnover associated with that variable, while holding all other variables constant. The shape of each curve indicates how the rate of genetic change in allele frequencies varies along the gradient. GEO: geographic distance; BIO7: temperature annual range (BIO5‐BIO6); BIO11: mean temperature of coldest quarter

For *E. pleiosperma* and *E. polyandra,* the GDM analysis explained, respectively, 63.90% and 81.35% of the deviance in spatial patterns of genetic (RAD‐SNP) turnover (both *P* values < 0.001), indicating good fit of the models to the data. There were seven variables (BIO4, BIO6, BIO7, BIO11, BIO14, BIO15, and GEO) that explained the observed genetic variation for *E. pleiosperma*, and the significant predictors were BIO7 (temperature annual range; importance weight = 0.63), GEO (geographic distance; importance weight = 0.48), and BIO11 (mean temperature of coldest quarter; importance weight = 0.41) (Table S4; Figure 5a). By contrast, for *E. polyandra*, GEO was the only significant predictor (Table S4; Figure 5b), indicating that its population genetic structure has a strong overall spatial component, driven by geographic isolation.

### 3.5 Signatures of climate‐driven adaptation in *E. pleiosperma*

**Figure 6:**
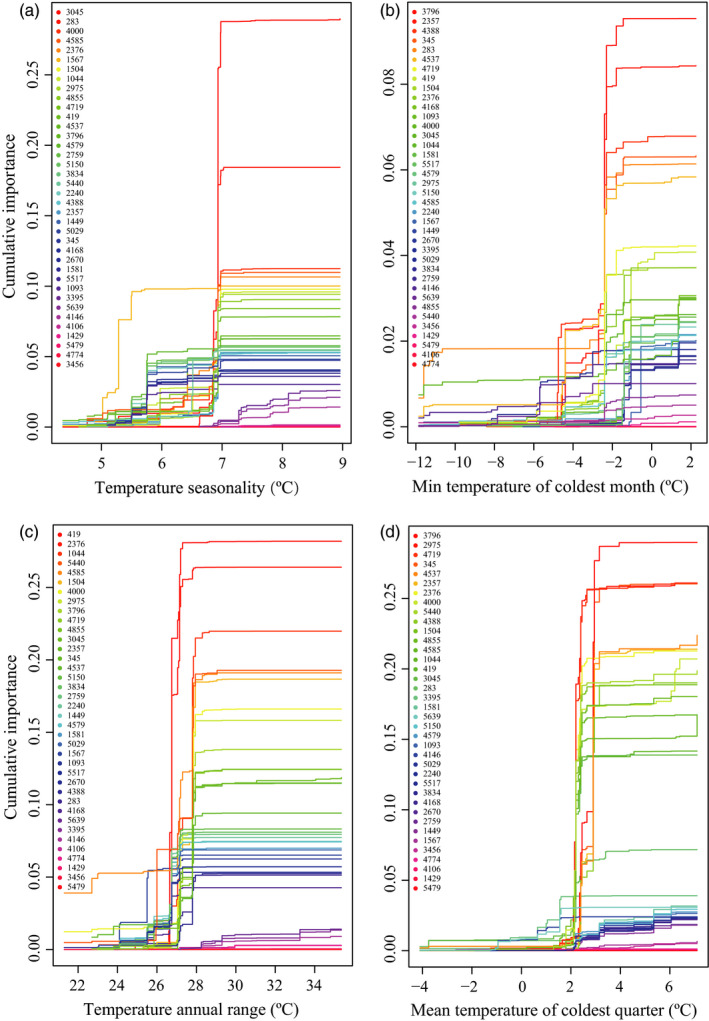
Gradient forest (GF) plots showing allelic turnover response curves of *F*_ST_ outliers (only shown for 38 loci with cumulative importance > 0) detected in *E. pleiosperma* in relation to (a) temperature seasonality (BIO4), (b) minimum temperature of the coldest month (BIO6), (c) temperature annual range (BIO7), and (d) mean temperature of the coldest quarter (BIO11). The key in the top left of each panel shows the loci with significant turnover in allele frequencies associated with each variable in order from high to low (top to bottom). The shape of each function indicates how the rate of change in allele frequencies varies along the gradient

Based on the entire filtered 8,782 SNPs of the “minimum” dataset, bayescan detected 203 outlier loci in *E. pleiosperma* and only one outlier in *E. polyandra*. Using arlequin, 368 outlier loci were identified in *E. pleiosperma* and four outlier loci were identified in *E. polyandra*. In *E. pleiosperma*, 49 loci were identified as *F*_ST_ outliers by both programs but none in *E. polyandra*. At these loci, the *SW* lineage showed much higher average within‐population genetic diversity than the *CE* lineage (*π* = 0.066/0.022, *H*_exp_ = 0.363/0.228, *H*_obs_ = 0.296/0.192). Across the species’ entire range, there was a significantly negative relationship between outlier‐derived *π* and longitude (*r* = −.14, *p = *.048; Figure 2d), but not for *π* and latitude or *H*_exp_/*H*_obs_ and longitude/latitude (all *p* values > .05). Based on the MLR analysis, only six outliers qualified as “adaptive” loci by showing *R* ^2^_adj_ values > 0.5 and significant correlations (*p* < .05) with four temperature‐related variables, including temperature seasonality (BIO4), minimum temperature of the coldest month (BIO6), temperature annual range (BIO7), and mean temperature of the coldest quarter (BIO11) (Table 4). Of the 49 outliers in *E. pleiosperma*, most SNPs showed the greatest allelic turnover magnitude at particular gradient positions with respect to BIO4 (highest allele turnover at *c.* 7°C; Figure 6a), BIO6 (highest allele turnover at *c.* −2°C, Figure 6b), BIO7 (highest allele turnover at between 26°C and 28°C, Figure 6c), and BIO11 (highest allele turnover at *c.* 2°C, Figure 6d). For BIO14 and BIO15, most SNPs showed weak allelic turnover magnitude at different gradient positions (data not shown).

**Table 4:** Results of outlier detection and environment–SNP association analyses on RAD data of *E. pleiosperma*

Outlier ID	*R* ^2^_adj_	Significant environmental variables
1871	0.65	BIO4**, BIO7**, BIO11*
3045	0.73	BIO4*
3395	0.57	BIO4*, BIO6**, BIO7**, BIO11*
4146	0.61	BIO4*, BIO6**, BIO7**, BIO11*
4537	0.56	BIO4*, BIO7*
5639	0.58	BIO4*, BIO6**, BIO7**, BIO11*

Note

BIO4: temperature seasonality; BIO6: minimum temperature of the coldest month; BIO7: temperature annual range; BIO11: mean temperature of coldest quarter.

** *p* < .01

* *p* < .05.

### 3.6 Genomic vulnerability prediction to future climate change

Under a model of future climate conditions for 2050, genomic vulnerability was predicted to be much higher for *E. pleiosperma* populations from central/east China compared to those from southwest China (i.e., in regions east versus west of the Sichuan Basin). By contrast, genomic vulnerability for *E. polyandra* was low across its range (Figure 6).

## 4 DISCUSSION

In this study, we applied stringent filtering methods to generate a high‐quality SNP dataset for phylogeographic inference. Consistent with previous results using nuclear microsatellites (440 samples and 8 microsatellites, Cao et al., 2016; 678 samples and 7 microsatellites, Wei et al., 2016), our analyses of RADseq datasets identified two classic phylogeographic breaks across the East China Sea and between the Sino‐Himalayan and Sino‐Japanese Forest subkingdoms (Cao et al., 2016; Wei et al., 2016). However, we were also able to detect three subclusters (*SW*1‒3) within the *SW* lineage of *E. pleiosperma* from our RADseq data (Figures S3b, S4b), suggesting that RADseq can recover finer population structure than microsatellites, despite including only 17.7‒38.6% of the individual samples in previous studies. In addition, we found that our inferences of population structure were relatively robust to missing data, at least when the percentage of missing data ranged from 49.7% (“maximum” dataset) to 93.6% (“minimum” dataset). Likewise, irrespective of whether or not we filtered out SNPs with MAF < 1%, our results recovered the same general phylogeographical pattern, suggesting that our inferences of population structure were less affected by minor allele frequency thresholds. Clearly, our RADseq datasets with fewer samples but many more loci recovered finer population structure and inferred a more detailed evolutionary history in *Euptelea*, when compared to microsatellites (Cao et al., 2016; Wei et al., 2016). Moreover, we were able to use the thousands of genomic SNPs contained in our RADseq datasets, along with environmental data, to provide insights into its local adaptation and genomic vulnerability to future climate change in this system.

### 4.1 Late Miocene speciation and diversification of *Euptelea*

Using ABC simulations, we dated the split between *E. pleiosperma* (China) and *E. polyandra* (Japan) at about the Late Miocene, *c*. 6.39 (95% CI: 3.90–8.92) Ma (Scenario 2; Figure 3a). This timing is very similar to our previous estimates inferred from fossil‐calibrated nuclear (26S nrDNA) (*c*. 5.46 Ma) and cpDNA phylogenies (*c*. 6.04 Ma) of *Euptelea* (Cao et al., 2016). Hence, the present results support our earlier vicariant‐speciation hypothesis for *Euptelea* (Cao et al., 2016). According to this, a Late Miocene landbridge across the East China Sea (ECS; *c.* 7.0–5.0 Ma; Kimura, 1996, 2003) would have allowed the common ancestor of *E. pleiosperma* and *E. polyandra* to migrate from China to Japan, followed by range fragmentation either due to an increasingly cooler and drier global climate around that time (Cerling & Sharp, 1996) and/or a subsequent rise in sea level (see also Cao et al., 2016). Concomitantly, all of our divergence time estimates, as well as species‐specific distribution models for the Last Glacial Maximum (LGM; *c.* 21,000 year before present, BP; Cao et al., 2016), dismiss the possibility of more recent speciation in *Euptelea* triggered by ECS landbridge submergence during the last glacial cycles. In support of this, all population‐based genetic data (cpDNA/nSSRs: Cao et al., 2016; RAD‐SNPs: this study) demonstrate the distinctiveness and long‐term isolation of *E. pleiosperma* and *E. polyandra*. A similar role of the glacially exposed ECS landbridge as migration “filter” has also been revealed in another Tertiary relict shrub species (*Platycrater arguta*; Qi, Yuan, Comes, Sakaguchi, & Qiu, 2014), whereas, for some tree species (e.g., *Cercidiphyllum japonicum*, *Kalopanax septemlobus* and *Quercus acuta*), the landbridge likely served as a migration corridor (Lee, Lee, Choi, & Choi, 2014; Qi et al., 2012; Sakaguchi et al., 2012). These contrasting biogeographical effects of the ECS landbridge as filter versus corridor likely reflect not only species‐specific habitat preferences but also other intrinsic biological features, especially recruitment properties (Cao et al., 2016; Qi et al., 2014).

### 4.2 Contrasting demographic histories between *E. pleiosperma* and *E. polyandra*

For *E. pleiosperma*, our best‐fitting ABC model (Scenario 2) identified the *SW* lineage as being ancestral to the *CE* group (Figure 3a)*.* Thus, the Hengduan Mt. Region (HMR) likely served as a source area for the species’ colonization of central/eastern China. In turn, this would suggest that its ancestral range initially retracted to southwest China (i.e., following the species’ origin) rather than occupying large portions of mainland (i.e., southwest and central/east) China, as depicted in the poor‐fitting vicariant scenario (“Scenario 4”; Figure S1). We dated the divergence of the *CE* group from within the *SW* lineage to the beginning of the Mid‐Pliocene, *c.* 3.96 (95% CI: 2.22–5.93) Ma (Figure 3a; Table 3). This timing coincides with the latest phase of intense uplifting of the HMR since the Late Miocene (Xing & Ree, 2017, and references therein). It is feasible, therefore, that the origin of the *CE* group reflects uplift‐driven population subdivision, divergence, and (incipient) speciation and associated changes in landscape and environmental conditions, as reported for numerous other plant and animal species from the HMR and adjacent areas (e.g., Fjeldså, Bowie, & Rahbek, 2012; He & Jiang, 2014; Xing & Ree, 2017; Yuan, Zhang, Peng, & Ge, 2008). Not unexpectedly, therefore, when compared with the *CE* group, the *SW* lineage features stronger genetic structure (AMOVA Table 1; DAPC Figure S3b), reflecting restricted dispersal in a topographically complex region of high mountains and deep river valleys (e.g., Fan et al., 2013).

Our estimated expansion time for the *CE* lineage, at approximately the Mid‐Pliocene [*c.* 3.43 (95% CI: 1.47–4.68) Ma; Table 3; Figure 3b], coincides well with the presumed time of connection between the ancient Upper Yangtze, which formerly drained into the South China Sea (Rüber, Britz, Kullander, & Zardoya, 2004), and the eastward flowing Middle/Lower Yangtze in the “Three Gorges Mt. Region” (TGMR; Liu et al., 2018; Zhang et al., 2016). This river capture event could have promoted the eastward expansion of *E. pleiosperma* out of the HMR. In support of this, we found a significant decrease of within‐population genetic diversity (in terms of *π*) with longitude (Figure 2a), that is, along the species’ presumed route of colonization and possibly as a result of serial founder events (Hewitt, 1999; Swaegers et al., 2014). Moreover, in the ML tree (Figure S6), all *CE* samples from east of the Sichuan Basin occupied a nested, and thus potentially derived, position relative to those originating from northwest (BM/LX) and southeast (HP) of the basin (i.e., in the HMR). In sum, therefore, the present data suggest that the *CE* lineage of *E. pleiosperma* originated in the HMR from within the *SW* lineage but then expanded its range eastward, predominantly along the Yangtze River valley and its tributaries. Similar scenarios of southwest‐to‐east migration have also been invoked for other tree species from China (e.g., *Sophora davidii*, Fan et al., 2013; *Myricaria laxiflora*. Liu, Wang, & Huang, 2009). Nonetheless, the two main lineages of *E. pleiosperma* (*SW* vs. *CE*) largely represent genetically cohesive units, with little evidence for admixture at RAD‐SNPs (except BM/LX/HP; Figure 1). When combined with the species’ overall strong population subdivision (*Φ*_ST_ = 0.44; Table 1), as also revealed by maternally inherited cpDNA (Cao et al., 2016), this would further suggest that both *SW* and *CE* populations largely persisted in separate and multiple MRF refugia over periods of Quaternary climate change, with only limited inter‐regional and population gene exchange via both pollen and seeds.

In contrast to *E. pleiosperma*, the genomic data of *E. polyandra* revealed markedly lower levels of population subdivision (*Φ*_ST_ = 0.13; Table 1). This could reflect, at least in part, a historical signature of the species’ latest range expansion. This was also confirmed by the fact that both Scenario 3 (“expansion‐shrinkage‐expansion”) and Scenario 1 (“expansion”) received substantial support when compared to Scenario 2 (“recent shrinkage”) in the ABC model selection. Specifically, Scenario 3 and Scenario 1 were consistent in showing an expansion of *E. polyandra* during the Pliocene, that is, *c.* 2.67 (95% CI: 0.83–4.65) Ma and *c.* 3.88 (95% CI: 0.71–9.21) Ma for Scenario 3 and Scenario 1, respectively. However, the ABC point estimates of expansion time and population size changes for *E. polyandra* have to be treated with caution, as our data could not provide reliable posterior distributions for these parameters due to lack of power (Figure 3 and Table S6), and likely overestimate the time of expansion. Despite these caveats, we propose that the true history of *E. polyandra* might be still included within the 95% credible interval. Supportive evidence for this relatively ancient expansion scenario comes from our previous analyses of the plastid sequence data with Bayesian skyline plots (BSPs), indicating that *E. polyandra* experienced a strong increase of *N*e from *c*. 0.5 Ma onwards (Cao et al., 2016). The population growth could well indicate that this moisture‐dependent island tree, such as *E. polyandra*, actually benefited from the intensification of the warm, wet summer monsoon in East Asia since the Mid‐Pleistocene (*c*. 1.0–0.78 Ma) (Han, Fang, & Berger, 2003). Notably, both RAD‐SNP data of this study and cpDNA data (Cao et al., 2016) provide no support for any extensive postglacial range expansion from the refugia at the coast areas of Japans’ Southeast Pacific Ocean, which was inferred by fossil pollen data (Gotanda & Yasuda, 2008) and ecological niche modeling (ENM) (Cao et al., 2016). Therefore, to provide accurate estimations of population sizes of *E. polyandra* at different times in the past, larger sample size and sequencing depth will be required in future studies.

### 4.3 Species and lineage differences in climate‐driven adaptation and genomic vulnerability

In addition to the history of divergence and demographic changes, one might expect that environmental factors have also contributed to the current genomic (RAD‐SNP) structure of *Euptelea*. Indeed, for *E. pleiosperma*, the GDM analysis indicated that both geography and two temperature‐related variables (BIO7, temperature annual range; BIO11, mean temperature of coldest quarter) shaped the spatial distribution of genomic variation in this species (Figure 5a). However, for *E. polyandra*, our GDM analysis only supported geographic distance as the main driving force of spatial genomic variation (Figure 5b). In addition, our population genomic data failed to detect outlier loci and strong population structure in *E. polyandra* (Figure 1b). This pattern most likely reflects a balance between gene flow and genetic drift across geographic space (Orsini, Vanoverbeke, Swillen, Mergeay, & De Meester, 2013; Wright, 1943).

In *E. pleiosperma*, of the 49 outlier loci identified by both *F*_ST_ outlier approaches, only six were inferred to be under diversifying selection using MLR analysis. They were significantly associated with four temperature‐related variables (BIO4, temperature seasonality; BIO6, min temperature of coldest month; BIO7, temperature annual range; and BIO11, mean temperature of coldest quarter) (Table 4), suggesting these regions of the genome are likely adaptive and diverge to a greater extent than the rest of the genome (Salojarvi et al., 2017). Nevertheless, due to the limited genomic resources available for *E. pleiosperma*, we cannot further annotate these six outlier loci, so their more specific role in local adaptation remains unclear. Nonetheless, across the species’ range, diversity at the 49 outlier loci (e.g., *π*) significantly decreased with longitude (Figure 2d), suggesting a functional role in climate adaptation. In addition, most of the 49 outlier loci showed a pronounced pattern of allelic turnover along the west‐to‐east gradients of temperature‐related variables (Figure 6). Together, these results provide compelling evidence that *E. pleiosperma* currently experiences climate‐driven diversifying selection.

**Figure 7:**
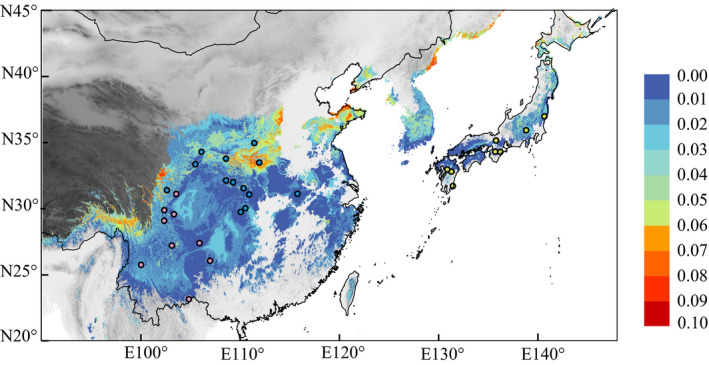
Map of genomic vulnerability across *E. pleiosperma* (China) and *E. polyandra* (Japan) distribution in the future (2050). Red = high genomic vulnerability; blue = low genomic vulnerability. The population locations (*n* ≥ 5) are represented by dots with color‐coding corresponding to the genetic structure in Figure 1

One may further expect that any sudden change in climate or climate variability will further increase the magnitude of diversifying selection (Exposito‐Alonso et al., 2018). In fact, by using climate projections for 2050, our metric of “genomic vulnerability” predicts that populations of the *CE* lineage are at the greatest risk of climate‐induced extinction, followed by those of the *SW* lineage, and *E. polyandra* (Figure 7). Based on our field investigations (Y.N. Cao, pers. obs.), *CE* populations are smaller and more fragmented than those from the other two regions. Further studies are required to test whether those populations from the Yangtze and its tributaries have already experienced some negative impacts of climate change over the past decades (see also Bay et al., 2018). Experimental studies across the *SW*/*CE* boundary could be highly informative in this regard, especially when combined with measures of allelic selection differentials linked to fitness and survival (Exposito‐Alonso et al., 2018; Linnen & Hoekstra, 2009).

## 5 CONCLUSIONS

To our knowledge, this study is the first that uses a multidisciplinary approach combining phylogenetics, phylogeography, and population‐ecological genomics to unravel the historical demography and climate‐related adaptation of an East Asian Tertiary relict genus, along with predictions about its “genomic vulnerability” to future climate change. The present data seem to suggest that the more complex geological history and greater environmental (e.g., physiographic, climatic) heterogeneity of subtropical China more readily promoted lineage diversification and a signal of adaptation in a Tertiary relict tree than was the case in Japan. This is evidenced by the deep intraspecific lineage divergence and the strong signal of local adaptation in *E. pleiosperma*, but not in *E. polyandra*. In fact, the major genetic groups identified in *E. pleiosperma* (*CE* vs. *SW*) that are adapted to different ecological niches and thus should be managed as two separate conservation units. As the locally adapted but genetically impoverished populations of the *CE* lineage are facing a particularly high risk of losing more genetic variation due to future climate change and habitat loss, conservation measures are urgently required. Broadly, therefore, our study highlights the significance of combining genomics with environmental data when assessing the impact of future warming on East Asia's Tertiary relict flora by quantifying the ecological factors that have produced genomic variation in this system.

## Supporting information

Supplementary Material

## Data Availability

Data for this study are available at Dryad Digital Repository: https://datadryad.org/review?doi=doi:10.5061/dryad.c77nm4g.
