# Supplementary material for: Genomic insights into historical population dynamics, local adaptation, and climate change vulnerability of the East Asian Tertiary relict Euptelea (Eupteleaceae)
Source: Evol Appl. 2020 Apr 13;13(8):2038–55. doi: 10.1111/eva.12960 (PMC7463308; doi:10.1111/eva.12960)
Supplement: Supplementary file 1 — Supplementary Material [file EVA-13-2038-s001.docx]

**Supporting information**

**Article title:** Genomic insights into historical population dynamics, local adaptation and climate change vulnerability of the East Asian Tertiary relict *Euptelea* (Eupteleaceae)

**Table S1** Geographic and genetic characteristics of the 35 Euptelea populations used for RADseq

| Populations | Locations | Longitude | Latitude | *n* | Neutral | | |  | Outlier | | |
| --- | --- | --- | --- | --- | --- | --- | --- | --- | --- | --- | --- |
|  |  |  |  |  | *π* | *H*_exp_ | *H*_obs_ |  | *π* | *H*_exp_ | *H*_obs_ |
| ***E. pleiosperma*** | | | | | | | | | | | |
| ***SW*** | | | | | | | | | | | |
| EM | Mt. Emei, Sichuan | 103.333 | 29.588 | 5 | 0.111 | 0.339 | 0.329 |  | 0.084 | 0.41 | 0.356 |
| QJ | Qiaojia, Yunnan | 103.115 | 27.215 | 5 | 0.101 | 0.365 | 0.362 |  | 0.033 | 0.262 | 0.2 |
| SM | Shimian, Sichuan | 102.338 | 29.094 | 5 | 0.109 | 0.333 | 0.315 |  | 0.055 | 0.308 | 0.257 |
| TQ | Mt. Erlangshan, Sichuan | 102.353 | 29.89 | 5 | 0.110 | 0.342 | 0.344 |  | 0.084 | 0.337 | 0.291 |
| YB | Yangbi, Yunnan | 100.017 | 25.751 | 5 | 0.075 | 0.405 | 0.389 |  | 0.029 | 0.467 | 0.333 |
| YN | Malipo, Yunnan | 104.829 | 23.156 | 5 | 0.053 | 0.431 | 0.590 |  | 0.037 | 0.35 | 0.45 |
| HS | Huishui, Guizhou | 106.968 | 26.066 | 6 | 0.089 | 0.336 | 0.326 |  | 0.084 | 0.377 | 0.2 |
| DF | Dafang, Guizhou | 105.903 | 27.407 | 5 | 0.098 | 0.345 | 0.320 |  | 0.092 | 0.339 | 0.217 |
| DY | Dujiangyan, Sichuan | 103.562 | 31.119 | 5 | 0.106 | 0.342 | 0.331 |  | 0.098 | 0.42 | 0.363 |
| **Mean** | － | － | － | － | **0.095** | **0.360** | **0.367** |  | **0.066** | **0.363** | **0.296** |
| ***CE*** | | | | | | | | | | | |
| LX | Lixian, Sichuan | 102.624 | 31.407 | 5 | 0.102 | 0.323 | 0.313 |  | 0.03 | 0.253 | 0.2 |
| BM^*^ | Pingwu, Sichuan | 104.431 | 32.681 | 4 | － | － | － |  | － | － | － |
| HP | Mt. Huping, Hunan | 110.531 | 30.039 | 5 | 0.093 | 0.357 | 0.410 |  | 0.105 | 0.224 | 0.236 |
| BT | Baotianman, Henan | 111.929 | 33.49 | 5 | 0.071 | 0.362 | 0.396 |  | 0 | 0 | 0 |
| DB | Mt. Daba, Chongqing | 108.58 | 32.141 | 5 | 0.091 | 0.312 | 0.309 |  | 0.032 | 0.298 | 0.12 |
| HX | Huxian, Shaanxi | 108.573 | 33.784 | 5 | 0.086 | 0.319 | 0.309 |  | 0.004 | 0.2 | 0.2 |
| KX | Kangxian, Gansu | 105.49 | 33.369 | 6 | 0.088 | 0.288 | 0.273 |  | 0.007 | 0.167 | 0.167 |
| LJ | Shennongjia, Hubei | 110.35 | 31.554 | 5 | 0.088 | 0.315 | 0.308 |  | 0 | 0 | 0 |
| SX | Xiaxian, Shanxi | 111.428 | 34.974 | 5 | 0.089 | 0.335 | 0.331 |  | 0.007 | 0.356 | 0.4 |
| TM^*^ | Mt. Tianmu, Zhejiang | 119.428 | 30.36 | 4 | － | － | － |  | － | － | － |
| TP | Mt. Tianping, Hunan | 110.062 | 29.758 | 5 | 0.064 | 0.383 | 0.370 |  | 0 | 0 | 0 |
| TS | Tianshui, Gansu | 106.107 | 34.303 | 5 | 0.089 | 0.317 | 0.297 |  | 0.048 | 0.467 | 0.04 |
| TT | Tiantangzhai, Anhui | 115.776 | 31.132 | 5 | 0.067 | 0.384 | 0.433 |  | 0.016 | 0.367 | 0.5 |
| YC | Dalaoling, Hubei | 110.92 | 31.08 | 5 | 0.088 | 0.345 | 0.345 |  | 0.008 | 0.356 | 0.4 |
| ZP | Zhenping, Shaanxi | 109.293 | 32.006 | 5 | 0.091 | 0.324 | 0.304 |  | 0.035 | 0.278 | 0.233 |
| **Mean** | － | － | － | － | **0.085** | **0.336** | **0.367** |  | **0.022** | **0.228** | **0.192** |
| **Mean_(species)_** | － | － | － | － | **0.089** | **0.346** | **0.350** |  | － | － | － |
| ***E. polyandra*** | | | | | | | | | | | |
| JC | Ju Chi County | 130.894 | 32.96 | 6 | 0.198 | 0.365 | 0.352 |  | － | － | － |
| NK | Nichinan-shi, Kyushu | 131.433 | 31.7 | 5 | 0.210 | 0.362 | 0.336 |  | － | － | － |
| MI | Takachiho-cho, Miyazaki | 131.27 | 32.797 | 5 | 0.201 | 0.363 | 0.350 |  | － | － | － |
| RY | Mima, Tokushima | 134.042 | 34.11 | 6 | 0.207 | 0.344 | 0.317 |  | － | － | － |
| DG^*^ | Daogen Prefecture | 132.819 | 35.182 | 3 | － | － | － |  | － | － | － |
| DQ^*^ | Daoqu Prefecture | 134.471 | 35.351 | 2 | － | － | － |  | － | － | － |
| TN | Tenkawa Village, Nara | 135.882 | 34.224 | 5 | 0.201 | 0.370 | 0.333 |  | － | － | － |
| NL | Nara Prefecture | 135.962 | 34.186 | 6 | 0.220 | 0.351 | 0.362 |  | － | － | － |
| KG | Kosaka, Gifu | 135.765 | 35.136 | 5 | 0.207 | 0.373 | 0.362 |  | － | － | － |
| SA^*^ | Chichibu, Saitama | 138.831 | 35.917 | 3 | － | － | － |  | － | － | － |
| IW | Iwaki, Fukushima | 140.664 | 36.927 | 5 | 0.179 | 0.383 | 0.374 |  | － | － | － |
| **Mean** (species) | － | － | － | － | **0.203** | **0.364** | **0.348** |  | － | － | － |

Included are the number of individuals genotyped at each population (*n*), the average nucleotide diversity (*π*), and the average expected/observed heterozygosity (*H*exp/*H*obs) for each population. Populations BM, TM, DG, DQ and SA, marked with asterisks, were not used for analyses of genetic diversity, GDM, GF and MLR (see text for further explanation).

**Table S2** Details of the parameter setting for dealing with RAD raw data

| Parameters | Description |
| --- | --- |
| third | [0] [assembly_name]: Assembly name. Used to name output directories for assembly steps |
| ./ | [1] [project_dir]: Project dir (made in curdir if not present) |
|  | [2] [raw_fastq_path]: Location of raw non-demultiplexed fastq files |
|  | [3] [barcodes_path]: Location of barcodes file |
| ./cyn/raw/R1/*_1.fq.gz | [4] [sorted_fastq_path]: Location of demultiplexed/sorted fastq files |
| denovo | [5] [assembly_method]: Assembly method (denovo, reference, denovo+reference, denovo-reference) |
|  | [6] [reference_sequence]: Location of reference sequence file |
| rad | [7] [datatype]: Datatype (see docs): rad, gbs, ddrad, etc. |
| AATTC, | [8] [restriction_overhang]: Restriction overhang (cut1,) or (cut1, cut2) |
| 0 | [9] [max_low_qual_bases]: Max low quality base calls (Q < 20) in a read |
| 33 | [10] [phred_Qscore_offset]: phred Q score offset (33 is default and very standard) |
| 6 | [11] [mindepth_statistical]: Min depth for statistical base calling |
| 6 | [12] [mindepth_majrule]: Min depth for majority-rule base calling |
| 10000 | [13] [maxdepth]: Max cluster depth within samples |
| 0.9 | [14] [clust_threshold]: Clustering threshold for de novo assembly |
| 0 | [15] [max_barcode_mismatch]: Max number of allowable mismatches in barcodes |
| 0 | [16] [filter_adapters]: Filter for adapters/primers (1 or 2=stricter) |
| 35 | [17] [filter_min_trim_len]: Min length of reads after adapter trim |
| 2 | [18] [max_alleles_consens]: Max alleles per site in consensus sequences |
| 5 | [19] [max_Ns_consens]: Max N's (uncalled bases) in consensus (R1, R2) |
| 5 | [20] [max_Hs_consens]: Max Hs (heterozygotes) in consensus (R1, R2) |
| 85/120/160 | [21] [min_samples_locus]: Min # samples per locus for output |
| 20 | [22] [max_SNPs_locus]: Max # SNPs per locus (R1, R2) |
| 5 | [23] [max_Indels_locus]: Max # of indels per locus (R1, R2) |
| 0.25 | [24] [max_shared_Hs_locus]: Max # heterozygous sites per locus (R1, R2) |
| 0 | [25] [edit_cutsites]: Edit cut-sites (R1, R2) (see docs) |
| 1, 2, 2, 1 | [26] [trim_overhang]: Trim overhang (see docs) (R1>, <R1, R2>, <R2) |
| * | [27] [output_formats]: Output formats (see docs) |
|  | [28] [pop_assign_file]: Path to population assignment file |

**Table S3** Summary statistics used in the present study for the ABC procedures

| **Single sample statistics** |
| --- |
| 1. Proportion of loci with null gene diversity (= proportion of monomorphic loci) |
| 2. Mean gene diversity across polymorphic loci (Nei, 1987) |
| 3. Variance of gene diversity across polymorphic loci |
| 4. Mean gene diversity across all loci |
| **Two sample statistics** |
| 1. Proportion of loci with null *F*_ST_ distance between the two samples |
| 2. Mean across loci of non-null *F*_ST_ distances between the two samples |
| 3. Variance across loci of non-null *F*_ST_ distances between the two samples |
| 4. Mean across loci of *F*_ST_ distances between the two samples |
| 5. Proportion of loci with null Nei’s distance between the two samples |
| 6. Mean across loci of non-null Nei’s distances between the two samples |
| 7. Variance across loci of non-null Nei’s distances between the two samples |
| 8. Mean across loci of Nei’s distances between the two samples |

**Table S4** The results of GDM analyses for *E. pleiosperma* and *E. polyandra*. The 19 bioclimatic variables downloaded from WorldClim (<http://www.worldclim.org>).

| Predictors | Code of predictors | Importance weight | |
| --- | --- | --- | --- |
|  |  | *E. pleiosperma* | *E. polyandra* |
| Geographic distance | GEO | **0.48^*^** | **0.1^*^** |
| Annual Mean Temperature | BIO 1 |  |  |
| Mean Diurnal Range [Mean of monthly (max temp-min temp)] | BIO 2 |  | 0.01 |
| Isothermality (BIO2/BIO7) (* 100) | BIO 3 |  |  |
| Temperature Seasonality (standard deviation *100) | BIO 4 | 0.17 |  |
| Max Temperature of Warmest Month | BIO 5 |  |  |
| Min Temperature of Coldest Month | BIO 6 | 0.28 | 0.01 |
| Temperature Annual Range (BIO5-BIO6) | BIO 7 | **0.63^*^** | 0.03 |
| Mean Temperature of Wettest Quarter | BIO 8 |  |  |
| Mean Temperature of Driest Quarter | BIO 9 |  |  |
| Mean Temperature of Warmest Quarter | BIO 10 |  |  |
| Mean Temperature of Coldest Quarter | BIO 11 | **0.41^*^** |  |
| Annual Precipitation | BIO 12 |  |  |
| Precipitation of Wettest Month | BIO 13 |  | 0.06 |
| Precipitation of Driest Month | BIO 14 | 0.08 |  |
| Precipitation Seasonality (Coefficient of Variation) | BIO 15 | 0.12 | 0.04 |
| Precipitation of Wettest Quarter | BIO 16 |  | 0.09 |
| Precipitation of Driest Quarter | BIO 17 |  |  |
| Precipitation of Warmest Quarter | BIO 18 |  |  |
| Precipitation of Coldest Quarter | BIO 19 |  |  |

* indicates the significant predictors in explaining the observed genetic variation in

| **Table S5** Description of RADseq data of *E. pleiosperma* and *E. polyandra*, showing individual codes, numbers of raw and filtered reads, cluster numbers, average depth, relative frequency of heterozygous loci (H) and error rate (E). | | | | | | | |
| --- | --- | --- | --- | --- | --- | --- | --- |
| Species/ individual code | Raw reads（×10^6^） | Filtered reads （×10^6^） | Clusters at 90%（×10^5^）^a^ | Average depth | *H* | *E* |  |
| ***E. pleiosperma*** | | | | | | |  |
| BM18 | 4.82 | 4.81 | 1.83 | 20.1 | 0.012 | 0.003 |  |
| BM2 | 7.06 | 7.06 | 2.27 | 24.11 | 0.014 | 0.003 |  |
| BM3 | 3.67 | 3.67 | 1.64 | 15.69 | 0.012 | 0.003 |  |
| BM8 | 8.89 | 8.88 | 2.21 | 30.65 | 0.012 | 0.003 |  |
| BT11 | 4.18 | 4.18 | 1.83 | 18.4 | 0.011 | 0.004 |  |
| BT3 | 11.43 | 11.42 | 2.55 | 36.38 | 0.014 | 0.003 |  |
| BT4 | 7.82 | 7.82 | 2.23 | 29.63 | 0.012 | 0.003 |  |
| BT5 | 2.49 | 2.48 | 1.36 | 13.02 | 0.012 | 0.004 |  |
| BT7 | 2.6 | 2.59 | 1.4 | 13.08 | 0.011 | 0.003 |  |
| DB18 | 5.26 | 5.25 | 2.02 | 19.72 | 0.012 | 0.007 |  |
| DB23 | 10.17 | 10.16 | 2.31 | 36.02 | 0.013 | 0.003 |  |
| DB25 | 7.07 | 7.06 | 2.18 | 25.69 | 0.013 | 0.003 |  |
| DB2 | 7.36 | 7.36 | 2.11 | 27.34 | 0.012 | 0.003 |  |
| DB7 | 13.89 | 13.87 | 2.39 | 46.24 | 0.014 | 0.003 |  |
| DF10 | 9.92 | 9.92 | 2.3 | 31.92 | 0.012 | 0.003 |  |
| DF15 | 3.68 | 3.68 | 1.6 | 16.99 | 0.012 | 0.004 |  |
| DF17 | 7.85 | 7.85 | 2.05 | 29.34 | 0.012 | 0.003 |  |
| DF1 | 5.05 | 5.03 | 2.05 | 18.71 | 0.011 | 0.007 |  |
| DF9 | 6.72 | 6.71 | 2.05 | 24.97 | 0.011 | 0.003 |  |
| DY13 | 5.12 | 5.12 | 2 | 18.04 | 0.012 | 0.003 |  |
| DY22 | 9.69 | 9.68 | 2.16 | 35.67 | 0.013 | 0.002 |  |
| DY2 | 7.7 | 7.69 | 2.15 | 28.29 | 0.012 | 0.003 |  |
| DY5 | 8.77 | 8.76 | 2.31 | 29.4 | 0.013 | 0.003 |  |
| DY7 | 8.92 | 8.92 | 2.25 | 31.28 | 0.013 | 0.003 |  |
| EM16 | 7.96 | 7.96 | 2.17 | 29.33 | 0.013 | 0.003 |  |
| EM19 | 7.99 | 7.99 | 2.12 | 29.69 | 0.012 | 0.003 |  |
| EM22 | 6.67 | 6.66 | 2.13 | 26.23 | 0.012 | 0.003 |  |
| EM26 | 11.58 | 11.57 | 2.31 | 37.69 | 0.013 | 0.003 |  |
| EM30 | 6.82 | 6.81 | 2.05 | 26.25 | 0.012 | 0.003 |  |
| HP12 | 6.7 | 6.68 | 2.17 | 24.54 | 0.012 | 0.007 |  |
| HP15 | 6.83 | 6.81 | 2.16 | 24.32 | 0.011 | 0.007 |  |
| HP16 | 6.77 | 6.75 | 2.27 | 23.47 | 0.013 | 0.007 |  |
| HP1 | 6.15 | 6.13 | 2.15 | 21.42 | 0.013 | 0.007 |  |
| HP20 | 7.39 | 7.36 | 2.32 | 25.78 | 0.013 | 0.007 |  |
| HS10 | 9.03 | 9.03 | 2.17 | 32.51 | 0.012 | 0.003 |  |
| HS13 | 5.38 | 5.38 | 1.84 | 22.56 | 0.011 | 0.004 |  |
| HS1 | 6.27 | 6.27 | 2.06 | 22.5 | 0.011 | 0.003 |  |
| HS4 | 6.12 | 6.12 | 1.98 | 23.98 | 0.011 | 0.003 |  |
| HS5 | 6.44 | 6.43 | 2.1 | 24 | 0.011 | 0.003 |  |
| HS7 | 8.28 | 8.28 | 2.16 | 28.99 | 0.012 | 0.004 |  |
| HX11 | 6.64 | 6.61 | 2.15 | 24.68 | 0.012 | 0.005 |  |
| HX14 | 6.41 | 6.41 | 1.99 | 25.95 | 0.012 | 0.004 |  |
| HX1 | 6.6 | 6.58 | 2.12 | 23.66 | 0.012 | 0.007 |  |
| HX4 | 11.07 | 11.03 | 2.35 | 36.7 | 0.013 | 0.005 |  |
| HX7 | 7.04 | 7.03 | 1.96 | 27.44 | 0.012 | 0.003 |  |
| KX13 | 3.71 | 3.7 | 1.79 | 16.28 | 0.011 | 0.005 |  |
| KX15 | 6.67 | 6.65 | 2.32 | 20.55 | 0.012 | 0.005 |  |
| KX17 | 6.71 | 6.69 | 2.22 | 23.66 | 0.012 | 0.005 |  |
| KX1 | 7.31 | 7.29 | 2.28 | 23.99 | 0.012 | 0.005 |  |
| KX8 | 7.95 | 7.94 | 2.17 | 29.08 | 0.012 | 0.003 |  |
| KX9 | 7.8 | 7.79 | 2.16 | 28.48 | 0.011 | 0.003 |  |
| LJ10 | 12.76 | 12.75 | 2.37 | 42.38 | 0.014 | 0.003 |  |
| LJ13 | 7.06 | 7.05 | 2.09 | 27.29 | 0.012 | 0.003 |  |
| LJ20 | 5.44 | 5.44 | 1.93 | 22.82 | 0.012 | 0.003 |  |
| LJ4 | 11.81 | 11.8 | 2.38 | 41.17 | 0.013 | 0.003 |  |
| LJ9 | 11.91 | 11.9 | 2.21 | 43.34 | 0.013 | 0.003 |  |
| LX1 | 16 | 15.99 | 2.48 | 53.44 | 0.014 | 0.002 |  |
| LX3 | 5.21 | 5.2 | 2 | 21.69 | 0.012 | 0.003 |  |
| LX5 | 6.39 | 6.39 | 1.97 | 24.4 | 0.013 | 0.003 |  |
| LX7 | 6.52 | 6.51 | 2.09 | 25.42 | 0.012 | 0.003 |  |
| LX9 | 2.5 | 2.5 | 1.35 | 13.83 | 0.013 | 0.003 |  |
| QJ1 | 7.22 | 7.22 | 2.04 | 26.94 | 0.012 | 0.003 |  |
| QJ3 | 8.39 | 8.39 | 2.13 | 30.05 | 0.012 | 0.003 |  |
| QJ5 | 6.96 | 6.95 | 2.01 | 27.1 | 0.012 | 0.003 |  |
| QJ7 | 9.02 | 9.02 | 2.23 | 32.54 | 0.012 | 0.002 |  |
| QJ9 | 3.79 | 3.79 | 1.49 | 18.75 | 0.013 | 0.003 |  |
| SM1 | 2.67 | 2.67 | 1.32 | 13.97 | 0.013 | 0.003 |  |
| SM3 | 3.6 | 3.6 | 1.62 | 16.21 | 0.013 | 0.003 |  |
| SM5 | 4.25 | 4.24 | 1.76 | 17.13 | 0.013 | 0.003 |  |
| SM7 | 4.03 | 4.02 | 1.74 | 17.53 | 0.013 | 0.003 |  |
| SM9 | 4.6 | 4.59 | 1.87 | 18.91 | 0.013 | 0.003 |  |
| SX10 | 4.8 | 4.8 | 2.03 | 18.96 | 0.011 | 0.003 |  |
| SX13 | 9.24 | 9.24 | 2.28 | 34.67 | 0.011 | 0.002 |  |
| SX1 | 9.87 | 9.87 | 2.13 | 37.5 | 0.012 | 0.002 |  |
| SX20 | 7.47 | 7.47 | 2.13 | 28.88 | 0.012 | 0.003 |  |
| SX5 | 10.33 | 10.32 | 2.29 | 37.34 | 0.012 | 0.002 |  |
| TM23 | 11.16 | 11.15 | 2.35 | 39.58 | 0.012 | 0.003 |  |
| TM25 | 4.94 | 4.94 | 2.05 | 20.4 | 0.01 | 0.003 |  |
| TM6 | 7.82 | 7.82 | 2.28 | 29.57 | 0.011 | 0.003 |  |
| TM8 | 6.45 | 6.44 | 2.18 | 24.79 | 0.011 | 0.003 |  |
| TP13 | 7.26 | 7.25 | 2.25 | 27.31 | 0.011 | 0.003 |  |
| TP16 | 12.03 | 12.02 | 2.31 | 42.03 | 0.011 | 0.003 |  |
| TP17 | 4.97 | 4.96 | 1.96 | 20.53 | 0.01 | 0.003 |  |
| TP18 | 1.63 | 1.63 | 0.86 | 11.17 | 0.012 | 0.005 |  |
| TP9 | 12.22 | 12.21 | 2.31 | 42.7 | 0.012 | 0.003 |  |
| TQ10 | 8.89 | 8.89 | 2.15 | 32 | 0.013 | 0.003 |  |
| TQ11 | 7.1 | 7.09 | 2.28 | 23.49 | 0.013 | 0.003 |  |
| TQ14 | 12.08 | 12.08 | 2.4 | 40.77 | 0.014 | 0.003 |  |
| TQ17 | 10.15 | 10.15 | 2.36 | 34.89 | 0.013 | 0.003 |  |
| TQ1 | 19.08 | 19.07 | 2.43 | 60.68 | 0.015 | 0.002 |  |
| TS14 | 5.11 | 5.11 | 2.06 | 18.31 | 0.012 | 0.003 |  |
| TS17 | 10.66 | 10.65 | 2.65 | 28.64 | 0.015 | 0.003 |  |
| TS3 | 9.12 | 9.11 | 2.3 | 28.66 | 0.014 | 0.003 |  |
| TS4 | 5.22 | 5.21 | 1.91 | 21.55 | 0.011 | 0.003 |  |
| TS9 | 11.43 | 11.42 | 2.67 | 32.82 | 0.015 | 0.003 |  |
| TT10 | 5.23 | 5.22 | 2.1 | 19.59 | 0.011 | 0.004 |  |
| TT13 | 9.51 | 9.5 | 2.24 | 34.24 | 0.011 | 0.002 |  |
| TT14 | 7.52 | 7.52 | 2.42 | 23.72 | 0.012 | 0.004 |  |
| TT2 | 9.73 | 9.72 | 2.32 | 33.98 | 0.012 | 0.003 |  |
| TT9 | 3.3 | 3.29 | 1.54 | 15.13 | 0.011 | 0.004 |  |
| YB12 | 9.23 | 9.23 | 2.46 | 29.94 | 0.011 | 0.002 |  |
| YB16 | 6.69 | 6.69 | 2.04 | 26.86 | 0.01 | 0.002 |  |
| YB3 | 8.1 | 8.09 | 2.11 | 31.2 | 0.011 | 0.002 |  |
| YB6 | 6.53 | 6.53 | 2.03 | 25.56 | 0.011 | 0.003 |  |
| YB9 | 6.39 | 6.39 | 2.07 | 26.43 | 0.011 | 0.002 |  |
| YC23 | 9.87 | 9.85 | 2.34 | 33.69 | 0.013 | 0.003 |  |
| YC2 | 12.14 | 12.13 | 2.41 | 38.57 | 0.013 | 0.003 |  |
| YC3 | 11.76 | 11.74 | 2.38 | 39.49 | 0.013 | 0.003 |  |
| YC5 | 9.71 | 9.7 | 2.43 | 33.73 | 0.013 | 0.003 |  |
| YC9 | 12.9 | 12.89 | 2.41 | 42.5 | 0.013 | 0.003 |  |
| YN13 | 3.99 | 3.99 | 1.57 | 18.7 | 0.012 | 0.004 |  |
| YN16 | 8.88 | 8.88 | 2.11 | 33.4 | 0.012 | 0.003 |  |
| YN19 | 10.2 | 10.2 | 2.21 | 35.55 | 0.012 | 0.003 |  |
| YN20 | 9.89 | 9.88 | 2.15 | 37.14 | 0.012 | 0.002 |  |
| YN7 | 7.88 | 7.87 | 2.03 | 30.72 | 0.01 | 0.003 |  |
| ZP13 | 10.63 | 10.62 | 2.35 | 34.77 | 0.013 | 0.003 |  |
| ZP16 | 9.76 | 9.75 | 2.33 | 32.32 | 0.012 | 0.003 |  |
| ZP3 | 9.86 | 9.85 | 2.14 | 35.99 | 0.012 | 0.003 |  |
| ZP6 | 11.4 | 11.39 | 2.34 | 38.02 | 0.013 | 0.003 |  |
| ZP7 | 11.06 | 11.04 | 2.36 | 38 | 0.013 | 0.003 |  |
| ***E. polyandra*** | | | | | | |  |
| DG6 | 12.78 | 12.78 | 2.96 | 34.25 | 0.014 | 0.002 |  |
| DG7 | 3.73 | 3.73 | 1.94 | 15.1 | 0.011 | 0.003 |  |
| DG9 | 11.1 | 11.09 | 2.42 | 37.3 | 0.012 | 0.002 |  |
| DQ11 | 8.84 | 8.83 | 2.36 | 33.37 | 0.012 | 0.002 |  |
| DQ5 | 9.17 | 9.16 | 2.35 | 35.01 | 0.012 | 0.002 |  |
| IW1 | 6.88 | 6.87 | 2.08 | 28.8 | 0.011 | 0.002 |  |
| IW2 | 5.17 | 5.17 | 2.28 | 15.14 | 0.012 | 0.004 |  |
| IW3 | 5 | 5 | 2.16 | 14.64 | 0.012 | 0.003 |  |
| IW5 | 4.62 | 4.62 | 1.89 | 18.99 | 0.011 | 0.003 |  |
| IW6 | 2.78 | 2.78 | 1.35 | 11.98 | 0.013 | 0.004 |  |
| JC10 | 6.11 | 6.1 | 2.03 | 25.72 | 0.011 | 0.003 |  |
| JC1 | 9.35 | 9.34 | 2.91 | 24.1 | 0.013 | 0.002 |  |
| JC3 | 5.44 | 5.43 | 2.11 | 20.4 | 0.012 | 0.005 |  |
| JC5 | 10.52 | 10.52 | 2.53 | 33.73 | 0.012 | 0.002 |  |
| JC7 | 12.31 | 12.3 | 2.61 | 35.97 | 0.013 | 0.002 |  |
| JC8 | 9.73 | 9.69 | 2.34 | 36.03 | 0.011 | 0.004 |  |
| KG10 | 8.17 | 8.17 | 2.61 | 23.69 | 0.013 | 0.003 |  |
| KG11 | 12.17 | 12.16 | 2.58 | 40.42 | 0.012 | 0.002 |  |
| KG1 | 13.56 | 13.55 | 3.26 | 31.67 | 0.016 | 0.003 |  |
| KG2 | 6.22 | 6.22 | 2.28 | 19.78 | 0.011 | 0.002 |  |
| KG9 | 9.18 | 9.17 | 2.25 | 35.98 | 0.011 | 0.002 |  |
| MI11 | 19.36 | 19.35 | 3.08 | 53.14 | 0.015 | 0.002 |  |
| MI12 | 9.41 | 9.41 | 2.39 | 33.67 | 0.012 | 0.002 |  |
| MI13 | 2.97 | 2.97 | 1.58 | 14.38 | 0.01 | 0.003 |  |
| MI4 | 9.09 | 9.09 | 2.66 | 27.38 | 0.013 | 0.002 |  |
| MI7 | 8.93 | 8.92 | 2.31 | 32.42 | 0.012 | 0.002 |  |
| NK14 | 6.47 | 6.47 | 2.05 | 26.93 | 0.011 | 0.002 |  |
| NK15 | 8.47 | 8.47 | 2.12 | 35.3 | 0.012 | 0.002 |  |
| NK1 | 3.93 | 3.93 | 1.64 | 14.18 | 0.012 | 0.003 |  |
| NK21 | 11.62 | 11.61 | 2.33 | 42.29 | 0.012 | 0.002 |  |
| NK26 | 10.99 | 10.98 | 2.88 | 32.04 | 0.013 | 0.002 |  |
| NL12 | 7.98 | 7.97 | 2.23 | 31.6 | 0.011 | 0.002 |  |
| NL13 | 7.02 | 7.02 | 2.09 | 28.72 | 0.012 | 0.002 |  |
| NL17 | 10.69 | 10.69 | 2.58 | 33.71 | 0.013 | 0.002 |  |
| NL1 | 10.32 | 10.32 | 2.27 | 38.66 | 0.013 | 0.002 |  |
| NL3 | 14.3 | 14.29 | 3.04 | 37.23 | 0.018 | 0.003 |  |
| NL4 | 6.95 | 6.94 | 2.27 | 23.32 | 0.012 | 0.003 |  |
| RY20 | 6.3 | 6.3 | 2.13 | 25.03 | 0.012 | 0.004 |  |
| RY22 | 9.58 | 9.57 | 2.21 | 38.28 | 0.01 | 0.002 |  |
| RY23 | 17.24 | 17.22 | 2.49 | 60.5 | 0.013 | 0.003 |  |
| RY2 | 4.97 | 4.97 | 1.98 | 21.38 | 0.012 | 0.004 |  |
| RY3 | 11.3 | 11.29 | 2.27 | 43.55 | 0.012 | 0.002 |  |
| RY4 | 15.39 | 15.38 | 2.36 | 55.18 | 0.013 | 0.002 |  |
| SA1 | 12.42 | 12.41 | 2.27 | 46.57 | 0.012 | 0.002 |  |
| SA6 | 7.44 | 7.44 | 2.2 | 30.38 | 0.011 | 0.002 |  |
| SA9 | 8.81 | 8.81 | 2.26 | 34.92 | 0.012 | 0.002 |  |
| TN1 | 3.87 | 3.87 | 1.63 | 16.8 | 0.011 | 0.003 |  |
| TN3 | 4.83 | 4.83 | 1.82 | 17.16 | 0.01 | 0.003 |  |
| TN5 | 8.39 | 8.38 | 2.07 | 22.9 | 0.012 | 0.003 |  |
| TN7 | 6.26 | 6.25 | 1.88 | 21 | 0.012 | 0.003 |  |
| TN8 | 9.08 | 9.08 | 2.14 | 24.2 | 0.012 | 0.002 |  |
| **Mean** | **8.09** | **8.08** | **2.16** | **28.78** | **0.012** | **0.003** |  |

^a^Clusters with more than the minimum depth of six reads. ^b^Consensus loci that passed filtering for paralogs.

| **Table S6** Cross-validation of parameter estimates for best-fit scenarios of *Euptelea*, each lineage of *E. pleiosperma* (*SW* and *CE*), and *E. polyandra* (*JP*) in Approximate Bayesian Computation analysis. Prediction error rate based on cross-validation were shown in the rejection, the local linear regression, and neural network methods with tolerance rates (0.001, 0.005, 0.01). | | | | | | | | | |
| --- | --- | --- | --- | --- | --- | --- | --- | --- | --- |
| Methods | Rejection | | | Local linear regression | | | Neuralnet | | |
| Tolerance rate | 0.001 | 0.005 | 0.010 | 0.001 | 0.005 | 0.010 | 0.001 | 0.005 | 0.010 |
| Divergence model (Scenario 2) | | | | | | | | | |
| NA | 0.198 | 0.223 | 0.254 | 0.113 | 0.069 | 0.072 | 0.270 | 0.139 | 0.104 |
| N(*SW*) | 0.124 | 0.127 | 0.135 | 0.031 | 0.027 | 0.030 | 0.034 | 0.083 | 0.103 |
| N(*CE*) | 0.153 | 0.152 | 0.166 | 0.053 | 0.073 | 0.078 | 0.133 | 0.206 | 0.194 |
| N(*JP*) | 0.132 | 0.149 | 0.198 | 0.046 | 0.037 | 0.038 | 0.094 | 0.075 | 0.064 |
| *t*_1_ | 0.051 | 0.069 | 0.083 | 0.036 | 0.051 | 0.046 | 0.269 | 0.152 | 0.124 |
| *t*_2_ | 0.325 | 0.310 | 0.354 | 0.207 | 0.172 | 0.172 | 0.295 | 0.373 | 0.357 |
| *SW* lineage (Scenario 1) | | | | | | | | | |
| Na | 0.286 | 0.267 | 0.198 | 0.171 | 0.172 | 0.239 | 0.296 | 0.291 | 0.281 |
| N1 | 0.185 | 0.193 | 0.297 | 0.045 | 0.038 | 0.036 | 0.216 | 0.213 | 0.202 |
| *t*_3_ | 0.184 | 0.184 | 0.293 | 0.082 | 0.089 | 0.230 | 0.205 | 0.208 | 0.232 |
| *CE* lineage (Scenario 1) |  |  |  |  |  |  |  |  |  |
| Na | 0.364 | 0.334 | 0.299 | 0.209 | 0.190 | 0.198 | 0.308 | 0.293 | 0.294 |
| N1 | 0.220 | 0.224 | 0.221 | 0.136 | 0.130 | 0.126 | 0.273 | 0.268 | 0.270 |
| *t*_3_ | 0.414 | 0.374 | 0.369 | 0.172 | 0.156 | 0.159 | 0.235 | 0.207 | 0.212 |
| *JP* lineage (Scenario 3) | | | | | | | | | |
| Na | 0.494 | 0.475 | 0.485 | 0.308 | 0.343 | 0.373 | 0.315 | 0.355 | 0.352 |
| N2 | 0.618 | 0.604 | 0.580 | 0.756 | 0.732 | 0.731 | 0.683 | 0.695 | 0.689 |
| Nb | 0.666 | 0.659 | 0.664 | 0.667 | 0.731 | 0.725 | 0.894 | 0.897 | 0.891 |
| N1 | 0.376 | 0.373 | 0.361 | 0.214 | 0.195 | 0.202 | 0.259 | 0.280 | 0.277 |
| *t*_3_ | 0.674 | 0.741 | 0.762 | 0.735 | 0.761 | 0.798 | 0.797 | 0.842 | 0.867 |
| *t*_b_ | 0.856 | 0.824 | 0.833 | 0.882 | 0.798 | 0.813 | 0.775 | 0.758 | 0.773 |
| *t*_4_ | 0.555 | 0.569 | 0.583 | 0.296 | 0.254 | 0.235 | 0.388 | 0.425 | 0.439 |
| *E. polyandra* (Scenario 1)* |  |  |  |  |  |  |  |  |  |
| Na | 0.544 | 0.555 | 0.632 | 0.384 | 0.404 | 0.459 | 0.811 | 0.782 | 0.758 |
| N1 | 0.351 | 0.332 | 0.308 | 0.249 | 0.241 | 0.225 | 0.350 | 0.360 | 0.367 |
| *t*_3’_ | 0.352 | 0.362 | 0.406 | 0.263 | 0.270 | 0.275 | 0.430 | 0.441 | 0.483 |


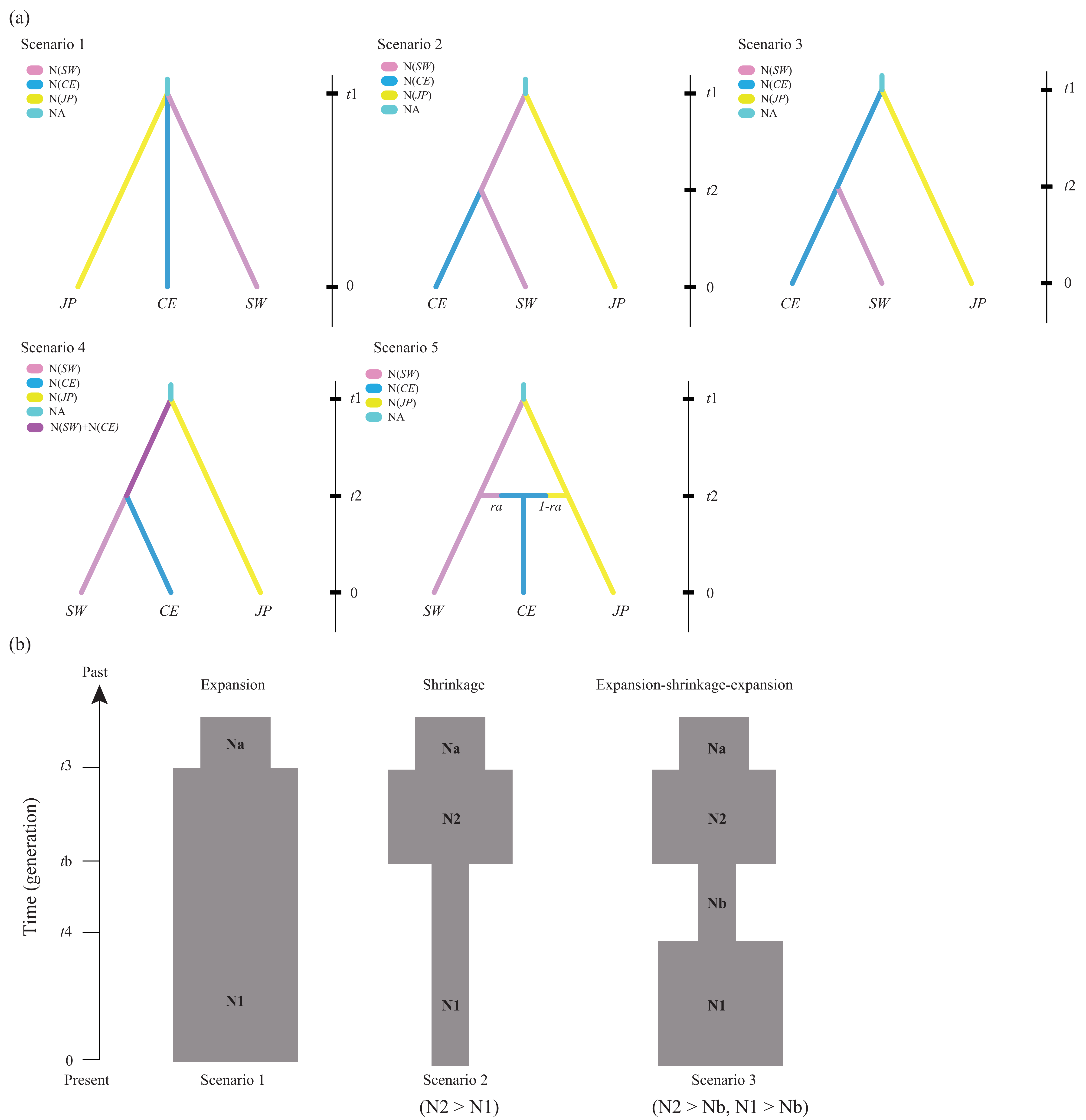


**Figure S1** (A) ABC models of divergence within *Euptelea*. *t*# = divergence time scaled by generation time. For scenario 1, *t*_1_ represents the trifurcate divergence time of the three groups. For scenarios 2‒4, *t*_2_ represents the divergence time between the *SW* and *CE* lineages, and *t*_1_ represents the divergence time between *E. pleiosperma* and *E. polyandra*. For scenario 5, *t*_1_ represents the divergence time between the *SW* lineage and *E. polyandra*, and *t*_2_ represents the time of genetic admixture between them, with *ra* being the admixture rate at time *t*_2_. Population sizes are marked in different colours. See Table 3 for all parameters in the best-fitting scenarios based on DIY-ABC. (B) Schematic representation of three demographic models (scenarios) of changes in population size. Na, ancestral population size; N1, current population size; N2 and Nb, population sizes between Na and N1; *t*_3_, old expansion time; *t*_b_, bottleneck time; *t*_4_, recent expansion time.


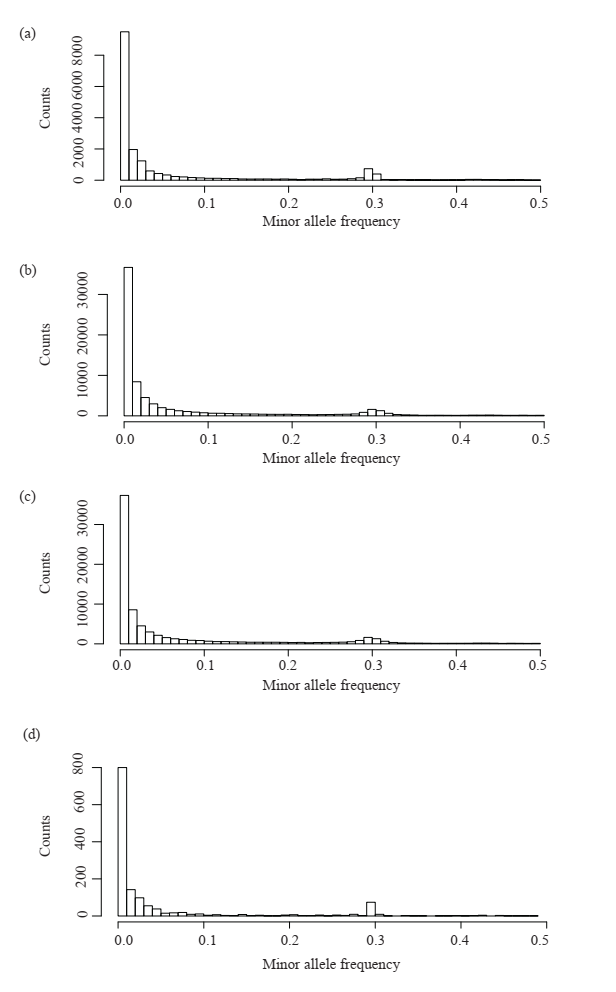


**Figure S2** Distribution of minor allele frequency for (a) ‘minimum’ dataset; (b) ‘median’ dataset, (c) ‘maximum’ dataset and (d) ‘full’ dataset.


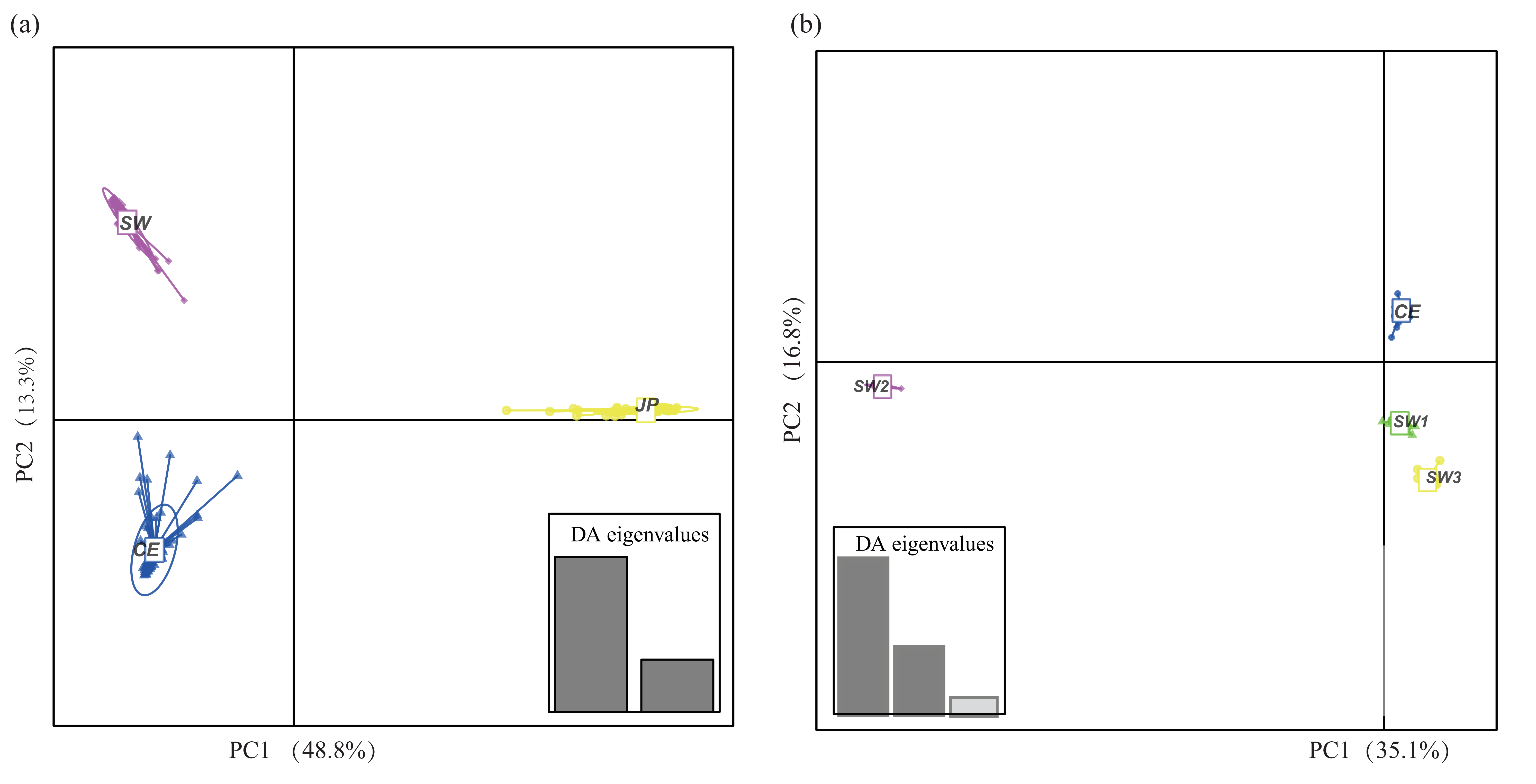


**Figure S3** Plots of the first two dimensions of discriminant analysis of principal component (DAPC) for (a) *Euptelea* with *K* = 3 and (b) *E. pleiosperma* with *K* = 4 based on the ‘maximum’, ‘median’ and ‘minimum’ datasets (data only shown for the ‘maximum’ dataset). *JP*, *E. polyandra*; *SW*, southwest China; *CE*, central/east China; *SW*1–3, the three subclusters of the *SW* lineage.


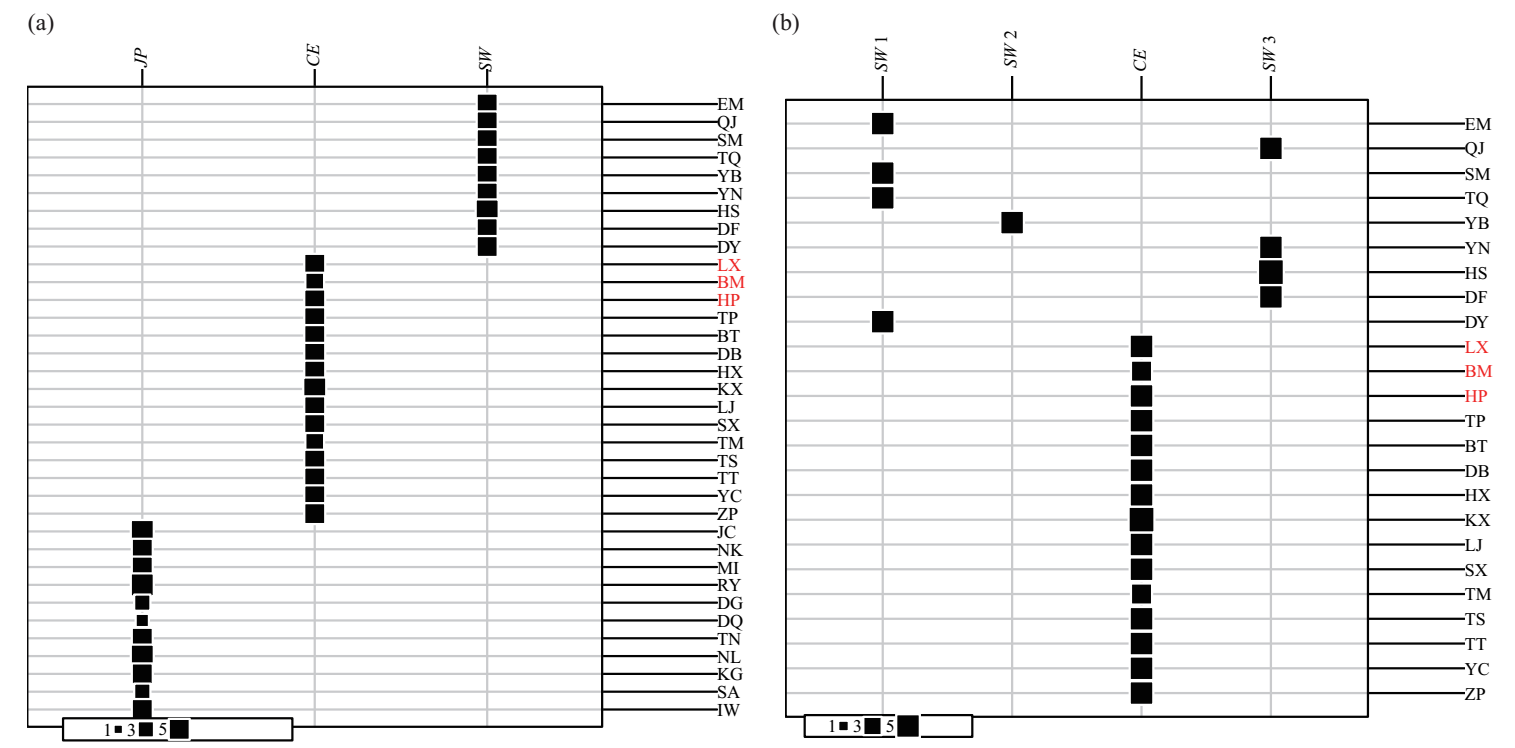


**Figure S4** Genetic grouping for (a) the 35 *Euptelea* populations with *K* = 3 and (b) 24 *E. pleiosperma* populations with *K* = 4 based on discriminant analysis of principal component (DAPC) of the ‘maximum’, ‘median’ and ‘minimum’ datasets (data only shown for the ‘maximum’ dataset).


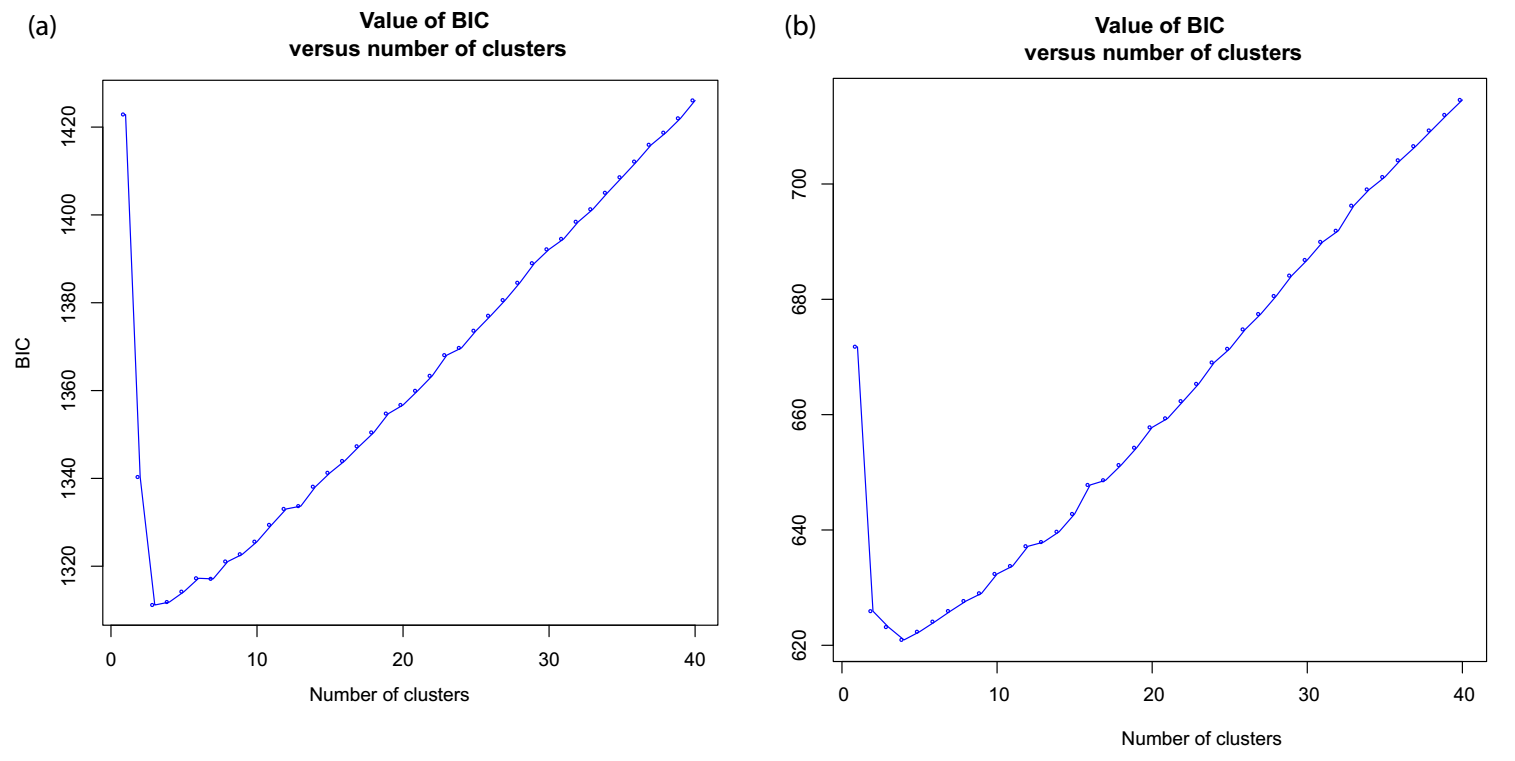


**Figure S5** The value of (Bayesian information criterion) for number of clusters in the discriminant analysis of principal component (DAPC) for (a) 35 *Euptelea* populations and (b) 24 *E. pleiosperma* populations based on the ‘maximum’, ‘median’ and ‘minimum’ datasets (data only shown for the ‘maximum’ dataset). Small dots on the curve represent the BIC values corresponding to each *K*, the lowest BIC means the optimal *K.*


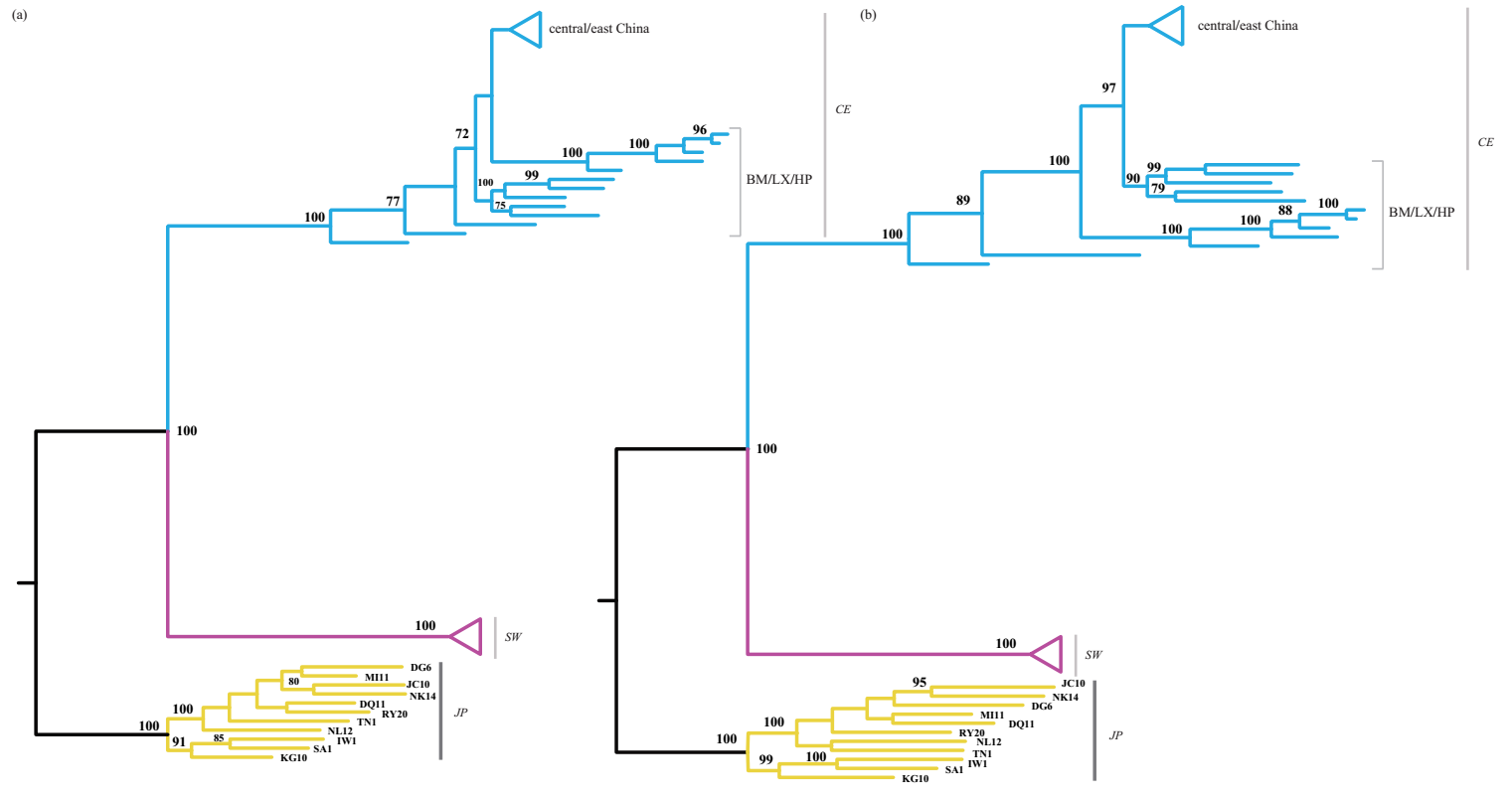


**Figure S6** Maximum likelihood (ML) phylogenetic tree inferred from (a) the ‘minimum’ dataset and (b) the ‘median’ and ‘maximum’ datasets (data only shown for the ‘maximum’ dataset) for 171 *Euptelea* individuals using raxml. Bootstrap percentage (BP) values (> 70%) are indicated above branches.


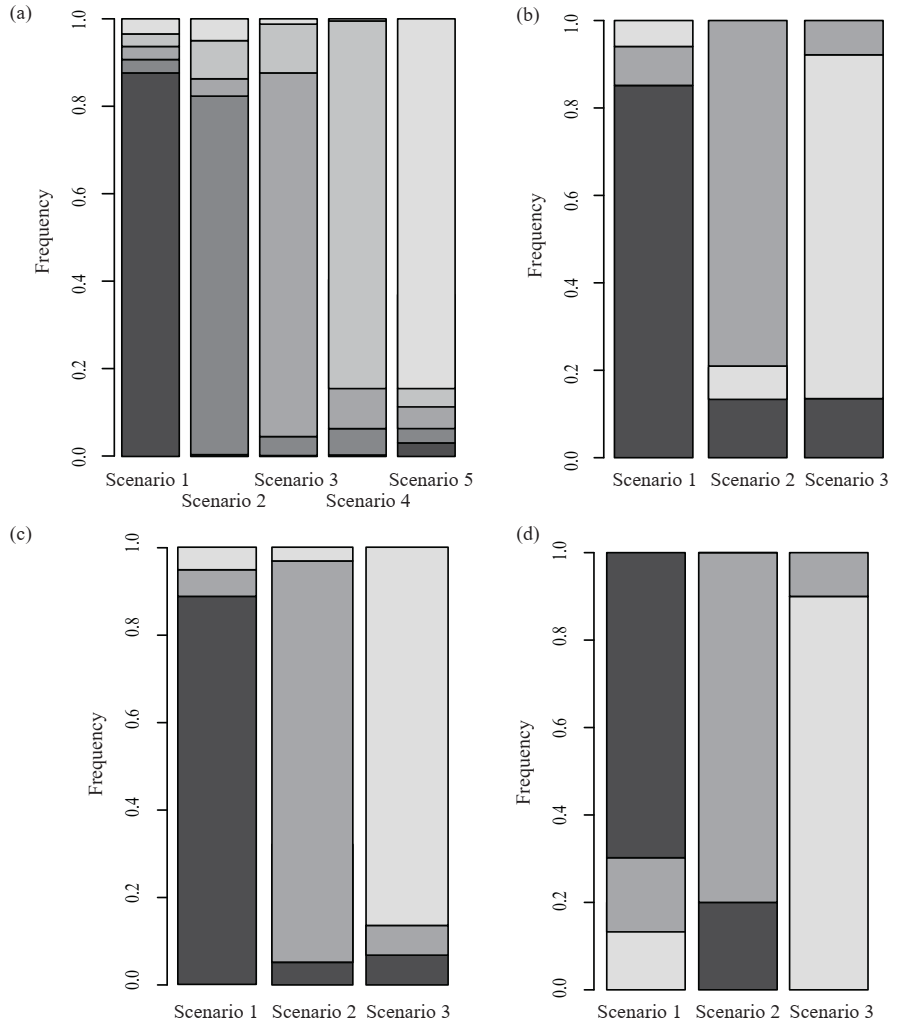


**Figure S7** Model misclassification for (a) divergence model and demographic model of (b) *SW* lineage, (c) *CE* lineage and (d) *JP* lineage via leave one out cross-validation using neural network method. The colors from dark to light grey correspond to each model from left to right, accordingly. If the simulations were perfectly classified, each bar would have a single color of its own corresponding model.


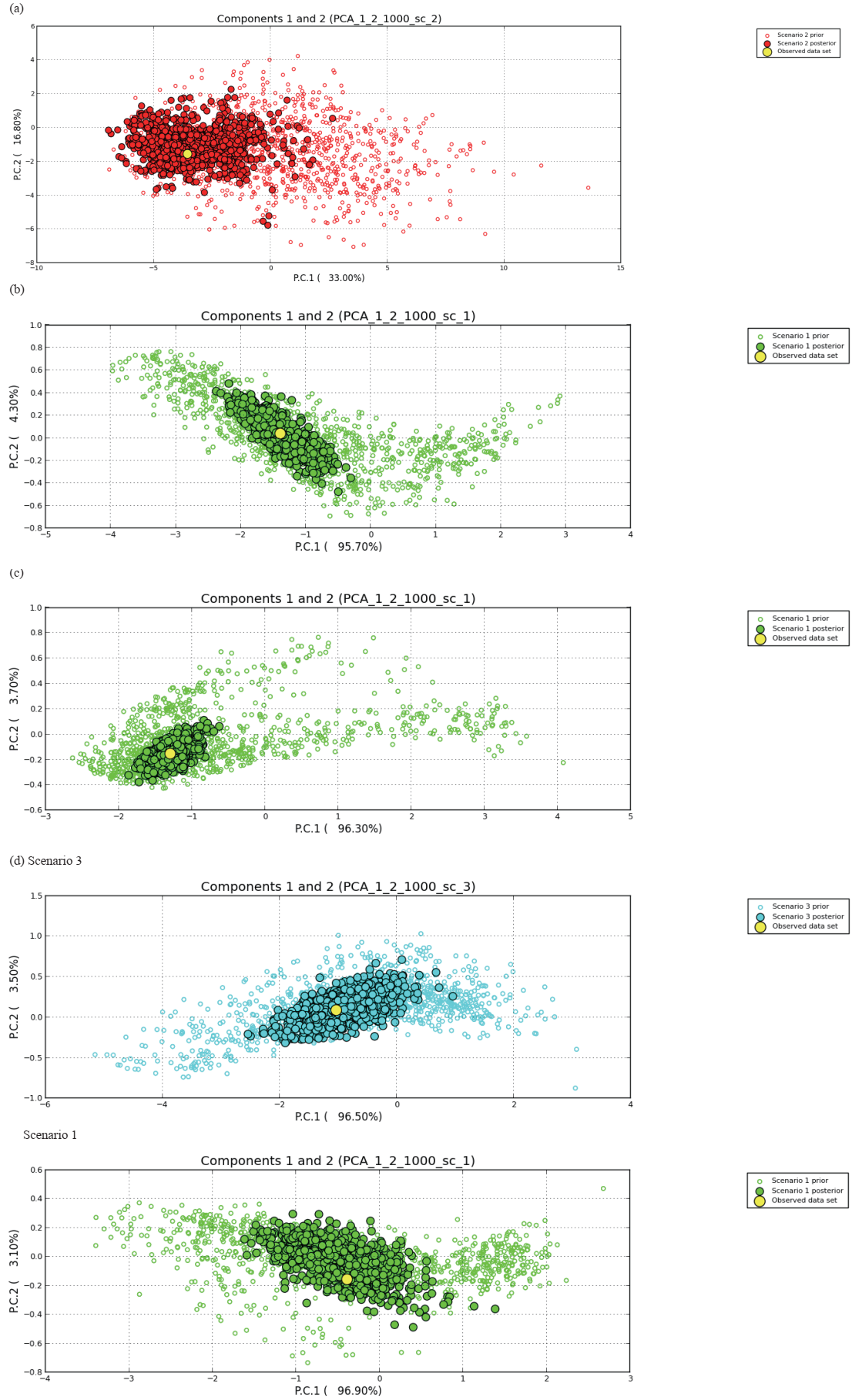


**Figure S8** Plots of principal component analysis (PCA) scores along the first two axes (PC1 and PC2) for (a) the ABC divergence analyses, and (b–d) the ABC demographic analyses of (b) the *SW* lineage of *E. pleiosperma*, (c) the *CE* lineage of *E. pleiosperma*, and (d) *E. polyandra*, as obtained from PCAs of summary statistics from the best-fitting scenarios. In the PCA, small open dots with coloration represent prior simulations, while the larger filled dots correspond to a dataset simulated with parameters drawn from the posterior distributions (1,000 datasets are randomly shown here). The large yellow dot corresponds to the real datasets for each species/lineage.
